# Chemotherapy‐Induced Senescence Reprogramming Promotes Nasopharyngeal Carcinoma Metastasis by circRNA‐Mediated PKR Activation

**DOI:** 10.1002/advs.202205668

**Published:** 2023-01-22

**Authors:** Qian Li, Yu‐Heng Zhao, Cheng Xu, Ye‐Lin Liang, Yin Zhao, Qing‐Mei He, Jun‐Yan Li, Kai‐Lin Chen, Han Qiao, Na Liu, Jun Ma, Lei Chen, Ying‐Qin Li

**Affiliations:** ^1^ Sun Yat‐sen University Cancer Center the State Key Laboratory of Oncology in South China Collaborative Innovation Center for Cancer Medicine Guangdong Key Laboratory of Nasopharyngeal Carcinoma Diagnosis and Therapy Center for Precision Medicine of Sun Yat‐sen University Guangzhou 510060 P. R. China; ^2^ Department of Radiation Oncology Sun Yat‐sen University Cancer Center Guangzhou 510060 P. R. China; ^3^ Department of Experimental Research Sun Yat‐sen University Cancer Center Guangzhou 510060 P. R. China

**Keywords:** circWDR37, metastasis, NF‐*κ*B, protein kinase R, senescence

## Abstract

Senescence is associated with tumor metastasis and chemotherapy resistance, yet the mechanisms remain elusive. Here, it is identified that nasopharyngeal carcinoma (NPC) patients who developed distant metastasis are characterized by senescence phenotypes, in which circWDR37 is a key regulator. CircWDR37 deficiency limits cisplatin or gemcitabine‐induced senescent NPC cells from proliferation, migration, and invasion. Mechanistically, circWDR37 binds to and dimerizes double‐stranded RNA‐activated protein kinase R (PKR) to initiate PKR autophosphorylation and activation. Independent of its kinase activity, phosphorylated PKR induces I‐kappaB kinase beta (IKK*β)* phosphorylation, binds to and releases RELA from NF‐*κ*B inhibitor alpha (I*κ*B*α*) to trigger nuclear factor kappa B (NF‐*κ*B) activation, thereby stimulating *cyclin D1* (*CCND1*) and senescence‐associated secretory phenotype component gene transcription in a circWDR37‐dependent manner. Low circWDR37 levels correlate with chemotherapy response and favorable survival in NPC patients treated with gemcitabine or cisplatin induction chemotherapy. This study uncovers a new mechanism of circWDR37 activated PKR in senescence‐driven metastasis and provides appealing therapeutic targets in NPC.

## Graphical abstract

Nasopharyngeal carcinoma (NPC) patients who developed distant metastasis are characterized by senescence phenotypes. A novel mechanism is further uncovered, in which circWDR37‐activated PKR promotes senescence‐driven metastasis through stimulating NF‐*κ*B signaling. CircWDR37 deficiency limits NPC cell metastasis and associates with chemotherapy benefits in NPC patients, thereby representing promising therapeutic strategy.

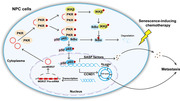

## 1 Introduction

Metastasis accounts for the leading cause of treatment failure in nasopharyngeal carcinoma (NPC).^[^ ^1^, ^2^ ^]^ Our phase III clinical trial showed that gemcitabine and cisplatin induction chemotherapy could eliminate micrometastases and prolong survival in patients with locoregionally advanced NPC.^[^ ^3^, ^4^ ^]^ However, patients with chemotherapy resistance succumb to metastasis, and the exact underlying mechanisms remain unclear. Emerging studies reveal that DNA damage induced by chemotherapy not only leads to cytotoxic death but also triggers cellular senescence in both primary and tumor cells.^[^ ^5^, ^6^ ^]^ Senescence is a multifaceted state in which cells undergo cell cycle arrest and exert suppressive or promoting effects on tumor development.^[^ ^7^, ^8^ ^]^ Recent findings suggest that chemotherapy‐induced senescence can promote metastasis via the deleterious effects of the senescence‐associated secretory phenotype (SASP) acquired by tumor cells.^[^ ^9^, ^10^, ^11^ ^]^ Therefore, inhibiting the detrimental effect of the SASP may help to prevent metastasis in senescent tumors following chemotherapy.

The secretion of proinflammatory cytokines, chemokines, and growth factors is the main characteristic of SASP.^[^ ^12^ ^]^ Nuclear factor kappa B (NF‐*κ*B) is a key molecular module involved in SASP generation.^[^ ^13^, ^14^ ^]^ Accumulating genomic studies have reported that somatic alterations and epstein‐barr virus (EBV) cooperate to sustain inflammatory NF‐*κ*B activation in NPC.^[^ ^15^, ^16^, ^17^ ^]^ Nonetheless, the somatic variation rate is relatively low for individual NPC patients, and the endogenous molecules sustaining constitutive NF‐*κ*B activation are not clear yet. Circular RNAs (circRNAs) are characterized by their structural covalent closed‐loop structure, and widespread among eukaryotes.^[^ ^18^, ^19^, ^20^ ^]^ They have been demonstrated to post transcriptionally mediate the immune response‐ and tumorigenesis‐related gene expression.^[^ ^21^, ^22^, ^23^ ^]^ Recent studies reveal that circRNAs are important in NF‐*κ*B signaling regulation.^[^ ^24^, ^25^ ^]^ However, much remains be elucidated about circRNAs involvement in SASP‐related NF‐*κ*B activation in NPC.

In this study, using global expression profiles, we identified senescence‐related phenotypes in distant metastatic NPC patients and circWDR37 as a key involved regulator. CircWDR37 deficiency sensitized NPC cells to chemotherapy‐induced senescence but inhibited metastasis. Mechanistically, circWDR37 interacts with double‐stranded RNA‐activated protein kinase R (PKR) to promote its homodimerization and phosphorylation and activates NF‐*κ*B signaling to induce SASP component gene transcription. Clinically, NPC patients with low circWDR37 expression benefit from induction chemotherapy. Taken together, our data reveal a novel mechanism underlying the chemotherapy‐induce senescence and metastasis, and suggest promising therapeutic targets in NPC.

## 2 Results

### 2.1 NPC Patients with Distant Metastasis Are Characterized by Senescence‐Related Phenotypes

**Figure 1:**
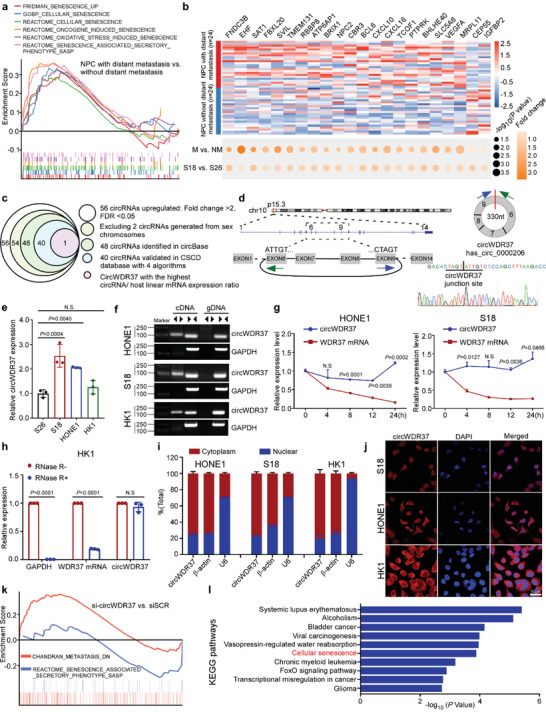
CircWDR37 is a key regulator in NPC senescence and metastasis. a) GSEA analysis of the microarray sequencing results of 24 paired locoregionally advanced NPC tissues with and without distant metastasis. b) Heat map of the indicated 22 senescence‐associated genes between 24 paired locoregionally advanced NPC tissues with and without distant metastasis. Bottom: Dot plot showing the −log_10_(*p*‐value) (circle size) and fold change (color scale) of each gene expression between 24 paired locoregionally advanced NPC tissues with or without distant metastasis (up) or S18 and S26 cell lines (down). c) Schematic illustration of identification of metastasis‐associated circRNAs in NPC. d) The genomic site of WDR37 gene. Blue and green arrows represent different primers. The effectiveness of reverse splicing joint is verified by Sanger sequencing. e) RT‐qPCR analysis of circWDR37 expression in S26, S18, HONE1, and HK1 cells. f) The expression of circWDR37 in cDNA and genomic DNA (gDNA). GAPDH was used as negative control. g) RT‐qPCR assay for the expression of circWDR37 and WDR37 mRNA in HONE1 and S18 cells treated with the transcription inhibitor Actinomycin D (2 µg mL^−1^) at the indicated time points. h) RT‐qPCR analysis of GAPDH, WDR37 mRNA, and circWDR37 expression after RNase R treatment in HK1 cells. i) RT‐qPCR analysis of subcellular circWDR37 expression in the nucleus and cytoplasm of HONE1, S18, and HK1 cells. *β*‐actin and U6 were used as endogenous controls. j) Subcellular localization of circWDR37 in HONE1, S18, and HK1 cells detected by RNA‐FISH. CircWDR37, red (Cy3); Nuclei, blue (DAPI). Scale bar, 50 µm. k) GSEA analysis of the RNA‐seq results of si‐circWDR37 and siSCR transfected S18 cells. l) KEGG pathways enrichment analysis in si‐circWDR37 target genes from RNA‐seq results. Mean (*n* = 3) ± s.d. (Data were analyzed by e) one‐way ANOVA with Dunnett's post‐hoc test, g) two‐way ANOVA with Bonferroni's post‐hoc test, and h) two‐tailed Student's *t*‐test). *p*‐value < 0.05 indicates statistical significance. N.S. indicates no significance.

To explore the contribution of senescence to tumor metastasis, we reanalyzed our previously published microarray transcriptome profiling data obtained from locoregionally advanced NPC tissues with (*n* = 24) and without distant metastasis (*n* = 24) after radical chemoradiotherapy.^[^ ^26^ ^]^ Gene set enrichment analysis (GSEA) revealed that senescence‐ and SASP‐associated signatures were significantly enriched in NPC patients with distant metastasis (**Figure** **1**a; Figure S1a, Supporting Information). Among the 137 differentially expressed mRNAs between NPC with and without distant metastasis (|fold change| > 1.5, *p* < 0.05), 22 were senescence‐ and SASP‐related signatures with 90.9% being significantly upregulated in NPC patients with distant metastasis (Figure 1b; Figure S1b, Supporting Information). The upregulated SASP signatures included those encoding growth factors and chemokines, such as C‐X‐C motif chemokine ligand 10 (CXCL10; fold change = 1.92847, *p* = 0.02894) and C‐X‐C motif chemokine ligand 16 (CXCL16; fold change = 1.80415, *p* = 0.00187). A principal component analysis (PCA) based on the expression of the 22 signatures revealed a distinction between NPC patients with and without distant metastasis (Figure S1c, Supporting Information).

To validate the expression pattern in NPC with metastasis, we employed a pair of NPC cell models with high (S18) and low (S26) metastatic abilities to conduct microarray expression profiling. As expected, S18 NPC cell showed similar expression alterations of senescence‐ and SASP‐related signature abundance (Figure 1b). Taken together, these results suggest that NPC patients with distant metastasis exhibit senescence features, and that genes involved in senescence may contribute to NPC metastasis.

### 2.2 CircWDR37 Is a Key Regulator in NPC Senescence and Metastasis

Emerging evidence demonstrates that circRNAs play critical roles in tumor progression and therapeutic resistance by directly or indirectly regulating gene expression. To investigate whether circRNAs participate in senescence associated metastasis in NPC, we analyzed the circRNA expression profiles in the S18 and S26 cell lines.^[^ ^27^ ^]^ Among the 56 circRNAs upregulated in S18 cell with high metastasis potential, 48 circRNAs were annotated in circBase database, and 40 were further validated with the cancer‐specific circRNA database (CSCD) via 4 different algorithms.^[^ ^28^ ^]^ Notably, hsa_circ_0000206, termed as circWDR37, showed the highest circRNA/host linear mRNA expression ratio among the circRNA candidates (Table S1, Supporting Information), which suggests the high expression level of circWDR37 relative to its linear WDR37 mRNA (Figure 1c).

CircWDR37 is head‐to‐tail spliced from the exons 6–9 of the *WDR37* gene; and the junction site was confirmed by Sanger sequencing (Figure 1d). Quantitative real‐time PCR (RT‐qPCR) analysis was performed to validate the expression of circWDR37 in NPC cells (S18, S26, HONE1, and HK1). The results confirmed that circWDR37 was significantly upregulated in S18 cells with high metastatic ability, when compared with that in S26 cells with low metastatic ability (*p* < 0.05, Figure 1e). Using reverse transcribed random primers, circWDR37 could be amplified with divergent primers in cDNA, but not in the corresponding genomic DNA (Figure 1f). After treatment with actinomycin D, the half‐life of circWDR37 expression was significantly longer than the linear WDR37 mRNA expression (*p* < 0.05, Figure 1g; Figure S1d, Supporting Information). Resistance to RNase R digestion further confirmed the closed loop structure of circWDR37 (Figure 1h; Figure S1e, Supporting Information). Nuclear and cytoplasm fractionation as well as fluorescence in situ hybridization (FISH) analysis showed that circWDR37 was mainly localized in cytoplasm (Figure 1i,j). These data demonstrate that circWDR37 is a bona fide circRNA upregulated in NPC cells with high metastatic potential.

To investigate the function of circWDR37, we first designed short interfering RNAs (siRNAs) targeting the backsplicing site of circWDR37 to specifically knock down circWDR37 expression (*p* < 0.05) without altering the linear WDR37 mRNA expression (Figure S1f, Supporting Information). Then, RNA sequencing (RNA‐seq) analysis was performed to examine the expression profiles of S18 cells with and without circWDR37 knocked down. The results identified 267 upregulated and 256 downregulated genes in S18 cells after circWDR37 knockdown (Figure S1g, Supporting Information). A GSEA analysis demonstrated significant enrichment of gene signatures associated with metastasis, SASP and nasopharyngeal carcinoma with circWDR37 expression (FDR < 0.05, Figure 1k; Figure S1h, Supporting Information). In addition, Kyoto Encyclopedia of Genes and Genomes (KEGG) analysis indicated that the differentially expressed genes were significantly enriched in the cellular senescence pathway (Figure 1l). Collectively, these findings indicate that circWDR37 is a key circRNA associated with senescence and metastasis in NPC.

### 2.3 CircWDR37 Deficiency Inhibits the Metastasis of Chemotherapy‐Induced Senescent NPC Cells In Vitro

**Figure 2:**
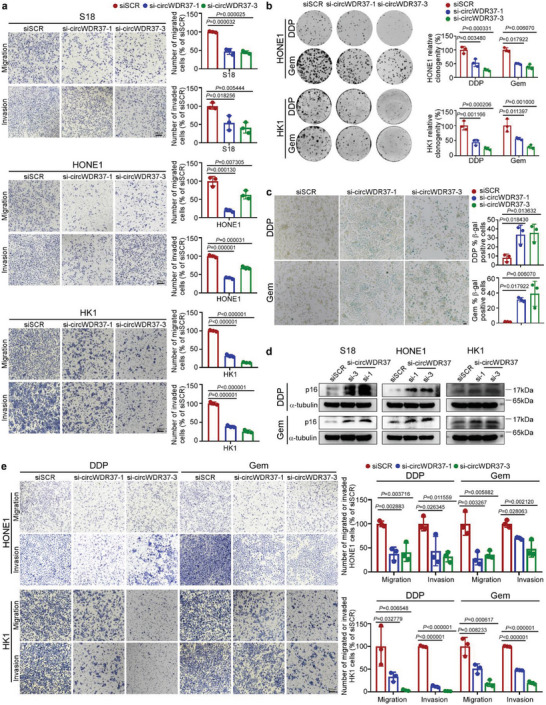
CircWDR37 deficiency inhibits the metastasis of chemotherapy‐induced senescent NPC cells in vitro. a) Representative images and quantified results of the transwell migration and invasion assays in S18, HONE1, and HK1 cells transfected with si‐circWDR37 or siSCR. b) Representative images and quantified results of the colony formation assay in si‐circWDR37 or siSCR transfected HONE1 and HK1 cells with cisplatin or gemcitabine treatment. DDP, Cisplatin; Gem, gemcitabine. c) Representative images and quantified results of SA‐*β*‐gal staining si‐circWDR37 or siSCR transfected HONE1 cells with cisplatin or gemcitabine treated for 24 h. d) Western blots analysis of senescence marker p16 in si‐circWDR37 or siSCR transfected HONE1, S18, and HK1 cells with cisplatin or gemcitabine treated for 24 h. e) Representative images and quantified results of the transwell migration and invasion assays in si‐circWDR37 or siSCR transfected HONE1 and HK1 cells with cisplatin or gemcitabine treated for 24 h. Mean (*n* = 3) ± s.d. (Data were analyzed by (a, b, c, e) one‐way ANOVA with Dunnett's post‐hoc test). *p*‐value < 0.05 indicates statistical significance. N.S. indicates no significance. Scale bar, 200 µm.

To validate the function of circWDR37 in NPC, we performed in vitro Transwell and senescence assays. Consistent with the GSEA results, circWDR37 knockdown significantly inhibited the migratory and invasive abilities of NPC cell lines (*p* < 0.05, **Figure** **2**a), and circWDR37 overexpression markedly promoted these phenotypes (*p* < 0.05, Figure S2a,b, Supporting Information), compared with the corresponding control. Next, we sought to determine whether circWDR37 affects the migration and invasion aggressiveness of senescent cells. Since cisplatin and gemcitabine are standard chemotherapy regimens for NPC and capable of inducing senescence,^[^ ^29^, ^30^, ^31^ ^]^ we treated NPC cells with cisplatin or gemcitabine to induce senescence and performed beta‐galactosidase (SA‐𝛽‐gal) staining, a standard cellular senescence marker. The results showed that circWDR37 depletion significantly reduced the cell growth (*p* < 0.05, Figure 2b) and increased SA‐𝛽‐gal expression in NPC cells treated with cisplatin or gemcitabine (Figure 2c; Figure S2c, Supporting Information). In addition, circWDR37 knockdown markedly enhanced the expression of p16^INK4a^ (cyclin‐dependent kinase 2a, CDKN2A), the most representative marker of cellular senescence, in NPC cells treated with cisplatin and gemcitabine (Figure 2d). Taken together, these data suggest that circWDR37 downregulation enhances cisplatin‐ or gemcitabine‐induced cellular senescence in NPC.

Although recent studies demonstrated that chemotherapy‐induced senescence promoted tumor metastasis through SASP,^[^ ^9^, ^10^, ^11^ ^]^ we were surprised to find that circWDR37 knockdown significantly restrained the migratory and invasive capabilities of NPC cells after senescence induction by cisplatin or gemcitabine (*p* < 0.05, Figure 2e). Together, these data reveal that circWDR37 deficiency promotes chemotherapy‐induced senescence but impairs the metastatic capability of senescent NPC cells in vitro.

### 2.4 CircWDR37 Stimulates NF‐*κ*B Activation to facilitate Chemotherapy‐Induced Proinflammatory SASP Gene Transcription

**Figure 3:**
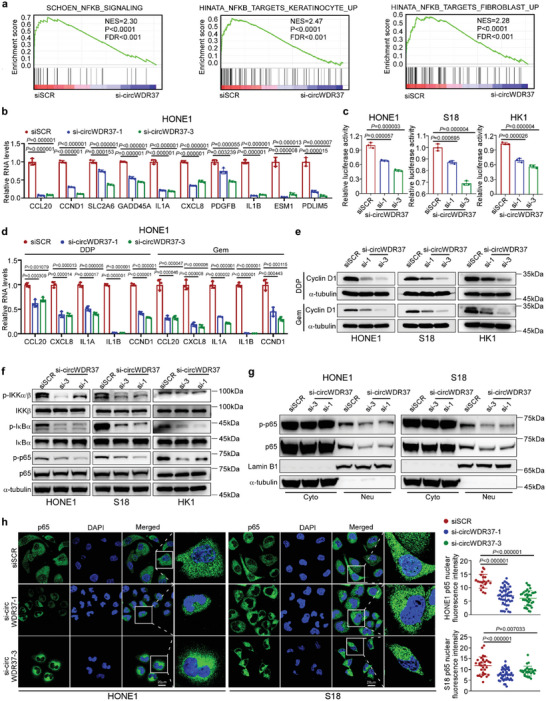
CircWDR37 stimulates NF‐*κ*B‐mediated chemotherapy‐induced proinflammatory SASP genes transcription. a) GSEA analysis of the RNA‐seq results of si‐circWDR37 and siSCR transfected S18 cells. NES, normalized enrichment score; FDR, false discovery rate. b) RT‐qPCR results for the mRNA expression of the indicated NF‐*κ*B‐targeted genes in HONE1 cells with circWDR37 knockdown. c) Luciferase assay of NF‐*κ*B activity in HONE1, S18 and HK1 cells transfected with si‐circWDR37 or siSCR. d) RT‐qPCR results for the mRNA expression of the SASP component in si‐circWDR37 or siSCR transfected HONE1 cells with cisplatin or gemcitabine treated for 24 h. e) Western blots analysis of cyclin D1 in si‐circWDR37 or siSCR transfected HONE1, S18, and HK1 cells with cisplatin or gemcitabine treated for 24 h. f) Western blots analysis of indicated proteins in HONE1, S18, and HK1 cells treated with si‐circWDR37or siSCR. g) Western blots analysis of the indicated proteins in cytoplasmic (cyto) and nuclear (neu) fractions of HONE1 and S18 cells transfected with si‐circWDR37 or siSCR. Lamin B1 and *α*‐tubulin were used as nuclear and cytoplasmic markers, respectively. h) Fluorescence microscopy analysis of HONE1 and S18 cells transfected with si‐circWDR37 or siSCR, p65 nuclear fluorescence intensity was determined by ImageJ software. Nuclei were stained with DAPI (blue). Scale bar, 20 µm. Mean (*n* = 3) ± s.d. (Data were analyzed by (b–d) one‐way ANOVA with Dunnett's post‐hoc test and (h) one‐way ANOVA with Scheffe's post‐hoc test). *p*‐value < 0.05 indicates statistical significance. N.S. indicates no significance.

We next elucidated the underlying mechanism by which circWDR37 regulates NPC senescence and metastasis. GSEA of the RNA‐seq transcriptome profiles showed that gene sets related to the NF‐*κ*B signaling pathway were significantly positively corelated with circWDR37 expression (FDR < 0.05, **Figure** **3**a; Figure S3a, Supporting Information). A panel of 18 NF‐*κ*B target genes were found to be downregulated in NPC cells with circWDR37 knockdown (Figure S3b, Supporting Information). The RT‐qPCR analysis validated that compared with siSCR control, circWDR37 knockdown significantly reduced the expression of 10 NF‐*κ*B target genes in HONE1, S18 and HK1 cells (*p* < 0.05, Figure 3b; Figure S3c,d, Supporting Information). The dual‐luciferase assays confirmed that circWDR37 depletion significantly inhibited the transcriptional activity of NF‐*κ*B in NPC cells (*p* < 0.05, Figure 3c).

The aforementioned downregulated genes included several cytokine‐ and chemokine‐encoding genes, such as interleukin 1 alpha (*IL1A*), interleukin 1 beta (*IL1B*), interleukin 8 (*IL8*, also known as *CXCL8*), and C‐C motif chemokine ligand 20 (*CCL20*), which are key proinflammatory SASP factors.^[^ ^7^ ^]^ Considering that the SASP is the most important feature of senescence and is critical for the senescence promoted metastasis,^[^ ^5^ ^]^ we further evaluated whether circWDR37 influence the SASP after chemotherapy induction. The results showed that circWDR37 deficiency indeed significantly inhibited the cisplatin‐ or gemcitabine‐induction of SASP acquisition, as shown by the decreased abundance of IL1A, IL1B, IL8, and CCL20 (Figure 3d; Figure S3e, Supporting Information). We also noticed that the expression of Cyclin D1 (CCND1), an NF‐*κ*B target gene, was remarkably decreased after chemotherapy at both mRNA and protein levels in NPC cells with circWDR37 knocked down (Figure 3d,e; Figure S3e, Supporting Information). CCND1, a p16 inhibitor, activates the cyclin‐dependent kinase 4/6 (CDK4/6), which induces cell cycle progression through the G1/S phase checkpoint to overcome cell cycle arrest,^[^ ^7^ ^]^ possibly explaining the mechanism through which circWDR37 deficiency sensitized NPC cells to cisplatin‐ or gemcitabine‐induced senescence through downregulating Cyclin D1 expression. These results indicate that circWDR37 depletion inhibits the chemotherapy‐induced proinflammatory SASP.

Phosphorylation of I‐kappaB kinase alpha/beta (IKK*α*/*β*), NK‐κB inhibitor alpha (I*κ*B*α*) and RELA (also known as p65) are the critical steps in the activation of classical NF‐*κ*B signaling.^[^ ^32^ ^]^ We found that circWDR37 knockdown significantly inhibited IKK*α*/*β*, I*κ*B*α*, and p65 phosphorylation without affecting their total protein expression (Figure 3f), and circWDR37 overexpression remarkably enhanced their phosphorylation (Figure S3f, Supporting Information). The phosphorylation and translocation of the NF‐*κ*B p50/p65 heterodimer into the nucleus contributes to target gene transcription.^[^ ^33^ ^]^ We found that the nuclear location of phosphorylated p65 was significantly reduced after circWDR37 knockdown (Figure 3g; Figure S4a, Supporting Information), whereas circWDR37 overexpression exerted the opposite effects (Figure S3g, Supporting Information). The immunofluorescence assay also confirmed the decreased nuclear translocation of p65 after circWDR37 knockdown (*p* < 0.05, Figure 3h; Figure S4b, Supporting Information). Collectively, these results reveal that circWDR37 activates NF‐*κ*B signaling to facilitate chemotherapy‐induced proinflammatory SASP component gene transcription in NPC cells.

### 2.5 CircWDR37 Induces PKR Homodimerization and Phosphorylation

**Figure 4:**
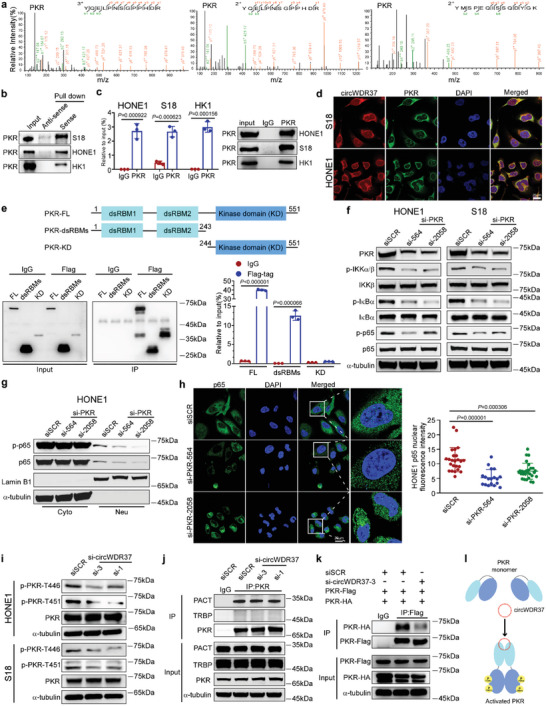
CircWDR37 induces PKR homodimerization and phosphorylation. a) Mass spectrometry identified PKR, which was pulled downed from S18 cells lysates by biotin‐labeled in vitro transcripted circWDR37. b) Representative western blots analysis of PKR pulled down by circWDR37. Sense: biotin‐labeled in vitro transcribed circWDR37; Anti‐sense: the corresponding complementary biotinylated RNA. c) Binding of circWDR37 to PKR in HONE1, S18, and HK1 cells were detected by RIP. d) Co‐localization of circWDR37 (red) with PKR proteins (green), respectively, in S18 and HONE1 cells. Scale bar, 20 µm. e) Binding of circWDR37 to the dsRBMs of PKR in HEK293T cells were detected by RIP. Top: a schematic diagram of PKR and its recombinant protein truncation proteins. f) Western blots analysis of the indicated proteins in HONE1 and S18 cells treated with si‐PKR or siSCR. g) Western blots analysis of the indicated proteins in cytoplasmic (cyto) and nuclear (neu) fractions of HONE1 cells transfected with si‐PKR or siSCR. Lamin B1 and *α*‐tubulin were used as nuclear and cytoplasmic markers, respectively. h) Fluorescence microscopy analysis of HONE1 cells transfected with si‐PKR or siSCR, p65 nuclear fluorescence intensity was determined by ImageJ software. Nuclei were stained with DAPI (blue). Scale bar, 20 µm. i) Western blot analysis of total PKR and phosphorylated PKR (p‐PKR‐T446, p‐PKR‐T451) in HONE1 and S18 cells treated with si‐circWDR37 or siSCR. j) Co‐IP showed the binding of PKR with PACT or TRBP proteins in HONE1 cells treated with si‐circWDR37or siSCR. k) Lysates from HONE1 cells cotransfected with PKR‐Flag and PKR‐HA along with si‐circWDR37 or siSCR were immunoprecipitated with anti‐Flag antibody, the precipitates and whole‐cell lysates were then analyzed by western blots with indicated antibodies. l) A schematic model for the function of circWDR37 in PKR activation. CircWDR37 physically interacts with PKR dsRBMs domain, resulting in homodimerization and autophosphorylation of PKR. Mean (*n* = 3) ± s.d. (Data were analyzed by (c, e) two‐tailed Student's *t*‐test, (h) one‐way ANOVA with Scheffe's post‐hoc test). *p*‐value < 0.05 indicates statistical significance. N.S. indicates no significance.

To investigate the mechanisms by which circWDR37 activates the NF‐*κ*B pathway, we performed an RNA pull‐down assay using in vitro‐transcribed circWDR37 with a biotin‐label, followed by mass spectrometry analysis. Among the pull‐downed proteins (Table S2, Supporting Information), circWDR37 was found to interact with double‐stranded RNA‐activated protein kinase R (PKR), a dsRNA sensor related to NF‐*κ*B activation (**Figure** **4**a; Figure S4c, Supporting Information).^[^ ^34^ ^]^ Western blot analysis following an RNA pull‐down assay further confirmed that the biotin‐labeled circWDR37 specifically bound to PKR in NPC cells, when compared with the antisense RNA (Figure 4b). The RNA immunoprecipitation (RIP) assay performed with anti‐PKR antibody revealed significant enrichment of circWDR37 (*p* < 0.05, Figure 4c). Consistently, immunofluorescence assay demonstrated that circWDR37 colocalized with PKR in the cytoplasm (Figure 4d; Figure S4d, Supporting Information). Next, to determine the domain critical for the binding of PKR to circWDR37, we generated a series of PKR deletion mutants and performed a RIP assay. A subsequent RT‐qPCR analysis showed that the dsRNA‐binding domains (dsRBMs) in the N‐terminal of PKR bound to circWDR37 as efficiently as the full‐length PKR (*p* < 0.05, Figure 4e), suggesting that the dsRBMs domain is critical for PKR binding to circWDR37.

It has been established that PKR can initiate NF‐*κ*B activation during infection and tumor promoting inflammation,^[^ ^35^, ^36^ ^]^ and we further evaluated whether PKR induces activation of the NF‐*κ*B cascade in NPC. The results demonstrated that PKR knockdown significantly inhibited the phosphorylation of IKK*α*/*β*, I*κ*B*α*, and p65 (Figure 4f; Figure S4e, Supporting Information). Moreover, the nuclear translocation of p65 was attenuated under PKR deficiency (Figure 4g,h; Figure S4f,g, Supporting Information), which was consistent with the effects of circWDR37 knockdown. Given that the activation of NF‐*κ*B cascade was induced by phosphorylated PKR,^[^ ^37^ ^]^ we further evaluated whether the effect of circWDR37 on NF‐*κ*B activation depends on PKR phosphorylation. Notably, the phosphorylation of PKR at Thr446 and Thr451, two phosphorylation sites crucial for PKR activation,^[^ ^38^ ^]^ was inhibited by circWDR37 silencing (Figure 4i). In contrast, forced circWDR37 expression induced PKR phosphorylation (Figure S4h, Supporting Information), suggesting that circWDR37 activates the NF‐*κ*B pathway through binding to PKR and inducing its phosphorylation.

We next explored how circWDR37 initiates PKR phosphorylation and activation. It has been reported that TAR RNA‐binding protein (TRBP) and protein activator of PKR (PACT) are two dsRNA‐binding proteins that exert the opposite regulatory effects on PKR.^[^ ^39^, ^40^ ^]^ Thus, we wondered whether circWDR37 affected the interaction between PKR and PACT/TRBP. However, no significant change was observed in the binding of PKR to PACT or TRBP after circWDR37 depletion (Figure 4j). Recent evidence demonstrated that long noncoding RNA bound to PKR at the dsRBMs, inducing PKR monomers to form homomers and subsequent phosphorylation.^[^ ^41^ ^]^ Then, we further investigated whether circWDR37 modifies the formation of PKR homodimerization. The results revealed that the interaction between HA and Flag‐tagged PKR was reduced by circWDR37 silencing (Figure 4k), suggesting that circWDR37 induces the homodimerization of PKR. Collectively, these results show that circWDR37 binds to PKR at the dsRBMs domain and induces PKR homodimerization and phosphorylation to activate NF‐*κ*B signaling (Figure 4l).

### 2.6 CircWDR37 Promotes PKR‐Stimulated IKK*β* Phosphorylation Independent of PKR Kinase Activity

**Figure 5:**
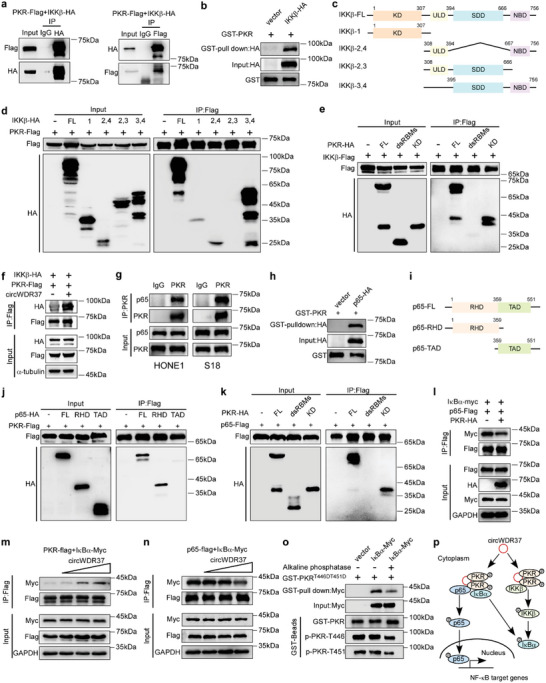
CircWDR37 promotes the binding of PKR to IKK*β* and facilitates the interaction of PKR with I*κ*B*α* thus releasing p65 from inhibitory I*κ*B*α*. a) Lysates from HEK293T cells co‐transfected with plasmids expressing PKR‐Flag, along with IKK*β*‐HA were immunoprecipitated with an anti‐HA antibody (left) or anti‐Flag antibody (right), the precipitates and whole‐cell lysates were then analyzed by western blots with indicated antibodies. b) In vitro purified GST‐PKR protein bound to agarose beads were added to the lysate of HEK293T cells overexpressing IKK*β*‐HA. c) The schematic diagram of IKK*β* and its recombinant protein truncation proteins. d–f) HEK293T cells transfected with the indicated plasmids were subjected to IP and then analyzed by western blots with indicated antibodies. g) Co‐IP showed the binding of endogenous PKR with p65 proteins in HONE1 and S18 cells. h) In vitro purified GST‐PKR protein bound to agarose beads were added to the lysate of HEK293T cells overexpressing p65‐HA. i) The schematic diagram of p65 and its recombinant protein truncation proteins. j–l) HEK293T cells transfected with the indicated plasmids were subjected to IP and then were analyzed by western blots with indicated antibodies. m) CircWDR37 (0, 1, 3, or 5 µg), PKR‐Flag and I*κ*B*α*‐myc were co‐transfected into HEK293T cells and processed for IP and were then analyzed by western blots with indicated antibodies. n) CircWDR37 (0, 1, 3, or 5 µg), p65‐Flag, and I*κ*B*α*‐myc were co‐transfected into HEK293T cells and processed for IP and were then analyzed by western blots with indicated antibodies. o) In vitro purified GST‐PKR^T446DT451D^ protein first treated with or without alkaline phosphatise, then bound to agarose beads and added to the lysate of HEK293T cells overexpressing I*κ*B*α*‐myc. p) A schematic model for the function of circWDR37 in PKR‐IKK*β* or PKR‐I*κ*B*α*‐p65 interaction. CircWDR37 promoted PKR interacting to IKK*β*. PKR and I*κ*B*α* competitively interacted with p65, which was also facilitated by circWDR37.

To address the regulatory effect of circWDR37 on PKR‐initiated NF‐*κ*B activation, we first conducted coimmunoprecipitation (co‐IP) assays to evaluate whether circWDR37 binds to NF‐*κ*B proteins. However, no direct interaction was observed between circWDR37 and IKK*β*, p65, or I*κ*B*α* (Figure S5a, Supporting Information). Then, we evaluated whether circWDR37 affects the binding of PKR to NF‐*κ*B components. First, the co‐IP assays demonstrated that the ectopically expressed PKR interacted with IKK*β* (**Figure** **5**a), and the glutathione‐S‐transferase (GST) pulldown assays confirmed their direct interaction (Figure 5b). To explore the regions critical for the PKR–IKK*β* interaction, we generated IKK*β* truncation mutants, including IKK*β*‐1(only with the kinase domain in the N‐terminal (KD)), IKK*β*‐2,4 (with a ubiquitination‐like domain (ULD) combined with a NEMO‐binding domain (NBD)), IKK*β*‐2,3 (with a ULD combined with a‐helical scaffold/dimerization domain (SDD) required for dimerization), and IKK*β*‐3,4 (with an SDD combined with an NBD) (Figure 5c). Further co‐IP assays revealed that IKK*β*‐2,4 and IKK*β*‐3,4 mutants, but not mutants without the NBD domain bound to PKR as efficiently as the full‐length of IKK*β* (Figure 5d), and that the KD domain of PKR specifically interacted with IKK*β* (Figure 5e). These results demonstrate that the interaction between IKK*β* and PKR specifically depends on the NBD domain of IKK*β* and the KD domain of PKR.

Next, we evaluated whether circWDR37 facilitates the interaction between PKR and IKK*β*. The co‐IP assay showed that the PKR binding to IKK*β* was potentiated after circWDR37 overexpression (Figure 5f). Although previous studies reported that PKR initiates NF‐*κ*B activation by phosphorylating IKK*β*, whether the regulatory mechanisms rely on PKR kinase activity remains unclear.^[^ ^42^, ^43^, ^44^ ^]^ Using in vitro kinase assays, we found that purified GST‐tagged PKR directly phosphorylated GST‐eukaryotic initiation factor 2 (eIF2*α*), a recognized PKR substrate,^[^ ^38^ ^]^ in a dose dependent manner, but did not phosphorylate GST‐IKK*β* (Figure S5b, Supporting Information). Altogether, the above findings suggest that circWDR37 promotes the binding of PKR to IKK*β*, which induces IKK*β* phosphorylation in a manner independent of PKR kinase activity.

### 2.7 CircWDR37‐Phosphorylated PKR Binds to and Releases p65 from the Inhibitory I*κ*B*α*

P65 complexed with p50 is the most abundant NF‐*κ*B protein.^[^ ^45^ ^]^ A previous study reported that PKR did not directly interact or phosphorylate p65.^[^ ^37^ ^]^ Surprisingly, we found that p65 phosphorylation was attenuated after PKR depletion in NPC cells (Figure 4f). Moreover, co‐IP assays revealed that PKR bound to p65 (Figure 5g), which was further confirmed by the GST pulldown assays (Figure 5h). To investigate the domains responsible for PKR‐p65 binding, we constructed p65 truncation mutants on the basis of its functional domains: the Rel homology domain (RHD) and the transcription activation domain (TAD) (Figure 5i).^[^ ^46^ ^]^ The co‐IP assays showed that the RHD domain in p65 bound to PKR as efficiently as the full‐length of p65, whereas the TAD domain showed no p65 binding capacity (Figure 5j). Moreover, the KD domain, but not the dsRBMs in PKR specifically interacted with p65 (Figure 5k).

Considering that the RHD domain is essential for p65 binding to the inhibitory protein I*κ*B*α*,^[^ ^24^ ^]^ we explored the impact of PKR on the interaction between p65 and I*κ*B*α*. Forced PKR expression significantly reduced the binding of I*κ*B*α* to p65 (Figure 5l), suggesting that PKR occupies the I*κ*B*α*‐binding site on p65. Moreover, we found that PKR physically interacted with I*κ*B*α* (Figure S5c, Supporting Information), which was reinforced by ectopic circWDR37 expression (Figure S5d, Supporting Information). These findings are consistent with a previous study showing that PKR interacted with and phosphorylated I*κ*B*α*.^[^ ^37^ ^]^ Next, we investigated whether circWDR37 affects the competition between PKR and I*κ*B*α* to bind p65. The increase in exogenous circWDR37 promoted the formation of the PKR/I*κ*B*α* complex (Figure 5m), and reduced the p65/I*κ*B*α* complex formation in a dose‐dependent manner (Figure 5n), implying that circWDR37 facilitates the binding of PKR to I*κ*B*α*, releasing p65 from the inhibitory I*κ*B*α* protein.

As circWDR37 initiated PKR phosphorylation and triggered NF‐*κ*B signaling, we further evaluated the role of circWDR37‐induced PKR phosphorylation on the PKR/I*κ*B*α* interaction. We constructed a mutant PKR by replacing the Thr446 and Thr451 residues with aspartic acid (T446DT451D), which led to constitutive phosphorylation. The in vitro purified PKR^T446DT451D^ proteins were preincubated with calf intestinal alkaline phosphatise, which forced their dephosphorylation, and then subjected to a GST pulldown assay. The results showed that PKR dephosphorylation reduced the PKR interaction with I*κ*B*α*, suggesting that the binding capability of PKR to I*κ*B*α* relies on PKR phosphorylation (Figure 5o). Collectively, these data illustrate that circWDR37‐phosphorylated PKR binds to and separates I*κ*B*α* from p65, and thereby releases p65 to translocate into the nucleus (Figure 5p).

### 2.8 PKR Is a Functional Mediator of circWDR37

**Figure 6:**
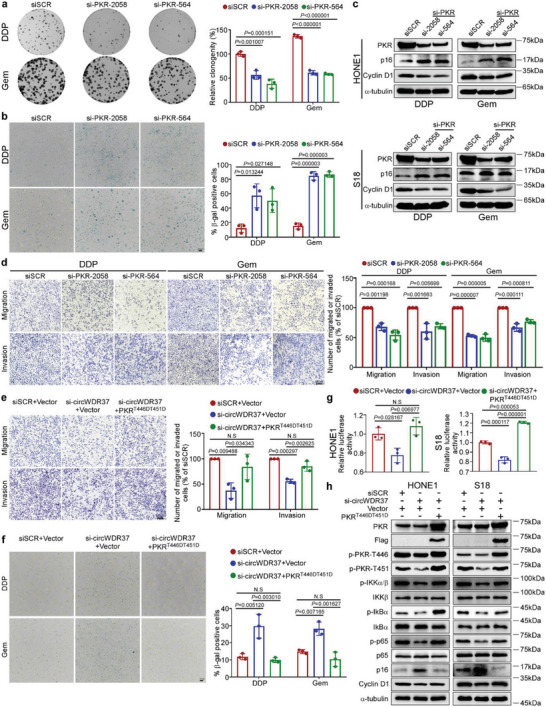
PKR is a functional mediator for circWDR37. a) Representative images and quantified results of the colony formation assay in si‐PKR or siSCR transfected HONE1 cells with cisplatin or gemcitabine treatment. b) Representative images and quantified results of SA‐*β*‐gal staining si‐PKR or siSCR transfected HONE1 cells with cisplatin or gemcitabine treatment. c) Western blots analysis of p16 and cyclin D1 in si‐PKR or siSCR transfected HONE1 and S18 cells with cisplatin or gemcitabine treatment. d) Representative images and quantified results of the transwell migration and invasion assays in si‐PKR or siSCR transfected HONE1 cells with cisplatin or gemcitabine treatment. e) Representative images and quantified results of transwell migration and invasion assays in the HONE1 cells co‐transfected with si‐circWDR37 or siSCR together with PKR^T446DT451D^‐Flag or empty vector. f) Representative images and quantified results of SA‐*β*‐gal staining in the HONE1 cells co‐transfected with si‐circWDR37 or siSCR together with PKR^T446DT451D^‐Flag or empty vector. g) Luciferase assay of NF‐*κ*B activity in HONE1 and S18 cells co‐transfected with si‐circWDR37 or siSCR together with PKR^T446DT451D^‐Flag or empty vector. h) Western blots analysis of the indicated proteins in HONE1 and S18 cells co‐transfected with si‐circWDR37 or siSCR together with PKR^T446DT451D^‐Flag or empty vector. Mean (*n* = 3) ± s.d. (Data were analyzed by (a, b, d) one‐way ANOVA with Dunnett's post‐hoc test and (e–g) one‐way ANOVA with Tukey's post‐hoc test). *p*‐value < 0.05 indicates statistical significance. N.S. indicates no significance. Scale bar, 200 µm.

The roles of PKR on NPC cell metastasis and senescence remain unknown. We next investigated the function of PKR in NPC cells. Similar to the effects of circWDR37 depletion, PKR knockdown remarkably suppressed the migration, invasion, and colony formation of NPC cells (*p* < 0.05, **Figure** **6**a; Figure S6a,b, Supporting Information). Moreover, PKR deficiency markedly increased SA‐𝛽‐gal‐stained NPC cells after cisplatin or gemcitabine treatment (*p* < 0.05, Figure 6b; Figure S6c, Supporting Information). Consistently, the expression of the senescence marker p16^INK4a^ was increased, whereas the Cyclin D1 expression was reduced by PKR depletion in cisplatin‐ or gemcitabine‐treated NPC cells (Figure 6c). These results confirm the inhibitory effects of PKR deficiency on NPC cell migration and invasion, and its promoting effects on cellular senescence. Moreover, the migration and invasion of chemotherapy‐induced senescent NPC cells were significantly impaired after PKR depletion (*p* < 0.05, Figure 6d). Taken together, these data indicate that PKR silencing exerts effects similar to those of circWDR37 knockdown, facilitating chemotherapy‐induced senescence of NPC cells and restricting their migration and invasion in vitro.

To confirm that circWDR37 antagonizes chemotherapy‐induced senescence and promotes NPC metastasis by activating PKR, we transfected NPC cells with circWDR37‐siRNA and PKR^T446DT451D^ overexpression plasmids. The attenuated migration and invasion of circWDR37‐depleted NPC cells were significantly reversed by persistent PKR phosphorylation at the T446 and T451 residues (*p* < 0.05, Figure 6e). Furthermore, ectopic PKR^T446DT451D^ expression markedly abrogated the enhanced chemotherapy‐induced cellular senescence induced by circWDR37 knockdown (*p* < 0.05, Figure 6f). Dual luciferase reporter assays and western blotting showed that exogenous PKR^T446DT451D^ manipulation markedly restored the inhibited NF‐*κ*B activation by circWDR37 depletion (Figure 6g,h). In additional, PKR^T446DT451D^ overexpression abolished the increase in senescence marker p16^INK4a^ expression and the reduced cyclin D1 expression in circWDR37‐knockdown cells (Figure 6h). These results reveal that PKR activation was critical for circWDR37‐mediated NPC cell senescence resistance, migration, and invasion.

### 2.9 CircWDR37 Deficiency Impairs NPC Cell Metastasis In Vivo

**Figure 7:**
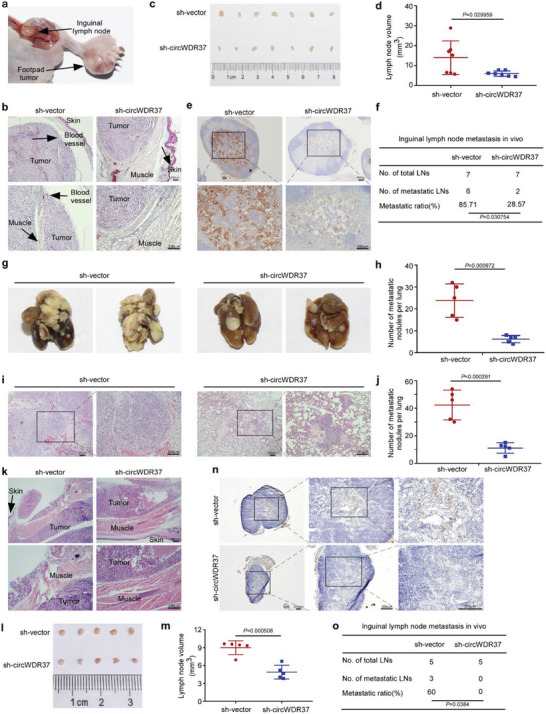
CircWDR37 deficiency impairs NPC cell metastasis in vivo. a) Representative images of footpad tumors after S18 cells with sh‐circWDR37 stable expression or its scramble control injected into the food pads of nude mice. b) Representative microscopy images of footpad tumors. c) Image and d) quantification of the volumes of the inguinal lymph nodes. e) Representative images of pan‐cytokeratin positive inguinal lymph nodes and f) inguinal lymph node metastatic ratios. Mean (*n* = 7) ± s.d. g) Representative images and h) quantification of macroscopic metastatic nodules on lung surfaces. Mean (*n* = 5) ± s.d. i) Representative images and j) quantification of microscopy metastatic nodules on lung tissues. Mean (*n* = 5) ± s.d. k) Representative microscopy images of footpad tumors. l) Image and m) quantification of the volumes of the inguinal lymph nodes. n) Representative images of pan‐cytokeratin positive inguinal lymph nodes and o) inguinal lymph node metastatic ratios. Mean (*n* = 5) ± s.d. (Data were analyzed by (d, h, j, m) two‐tailed Student's *t*‐test and (f, o) Chi‐square test). *p*‐value < 0.05 indicates statistical significance. Scale bar, 200 µm.

To validate the function of circWDR37 in vivo, we first generated an inguinal lymph node metastasis model. S18 cells with stable circWDR37 knockdown or a scramble control were transplanted into the foot pads of nude mice (Figure S7a,b, Supporting Information). After 4 weeks, the primary tumors on the foot pads and inguinal lymph nodes were dissected (**Figure** **7**a). In the circWDR37‐knockdown group, the footpad tumors were less aggressive, with relatively less invasion into the skin and muscle, as seen by hematoxylin‐eosin (H&E) staining (Figure 7b). Notably, circWDR37 depletion significantly reduced the tumor volumes (*p* < 0.05, Figure 7c,d) and the metastatic ratios in inguinal lymph nodes, as determined by the pancytokeratin staining (*p* < 0.05, Figure 7e,f). Next, S18 cells with stable circWDR37 depletion or a scramble control were injected into nude mice via the tail vein to establish a lung metastatic colonization model. As shown in Figure 7g,h, circWDR37 depletion markedly reduced the number of lung metastatic nodules in vivo (*p* < 0.05). In addition, H&E staining revealed that circWDR37 inhibition led to significantly fewer and smaller lung metastasis nodules (*p* < 0.05, Figure 7i,j). Together, these results confirm that circWDR37 knockdown suppresses NPC cell metastasis in vivo.

We further used the inguinal lymph node metastasis model to investigate the effect of circWDR37 on NPC cell metastasis upon treatment with cisplatin and gemcitabine. The mice were intraperitoneally injected with cisplatin (5 mg kg^−1^) and gemcitabine (50 mg kg^−1^) every 3 days after tumor establishment. Compared with the sh‐vector control group, circWDR37 knockdown inhibited the aggressive invasion toward skin and muscle of the footpad tumors after treated with cisplatin and gemcitabine (Figure 7k). Moreover, the volumes and metastatic ratios of the inguinal lymph nodes were significantly decreased in the circWDR37‐depleted group upon cisplatin and gemcitabine treatment (*p* < 0.05, Figure 7l–o). All together, these data demonstrate that NPC cells with circWDR37 knockdown could gain metastasis control benefit from cisplatin and gemcitabine treatment.

### 2.10 Low circWDR37 Expression Is Associated with a Favorable Prognosis and Better Response to Induction Chemotherapy in NPC Patients

**Figure 8:**
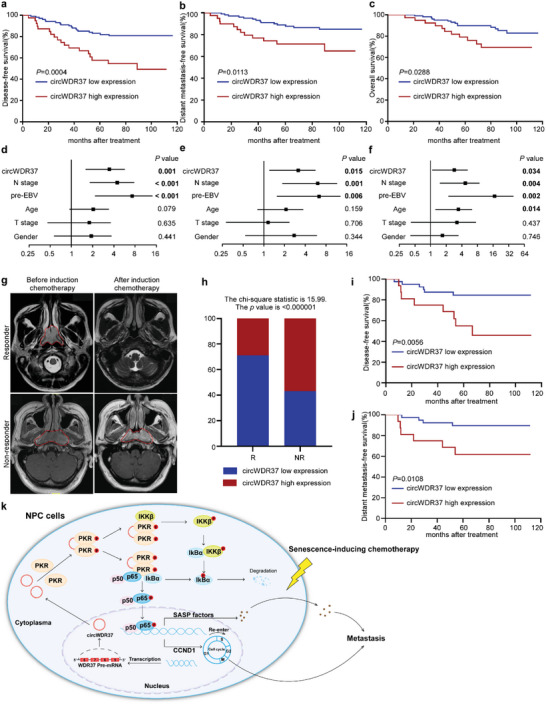
CircWDR37 high expression is associated with an adverse prognosis and a lack of responsiveness to induction chemotherapy in NPC patients. a) Kaplan–Meier analysis of disease‐free survival, b) distant metastasis‐free survival, and c) overall survival in patients with low circWDR37 expression (*n* = 39) and high circWDR37 expression (*n* = 101) groups. Forest plots of univariate analysis showing the significance of d) different prognostic variables in disease‐free survival, e) distant metastasis‐free survival, and f) overall survival of NPC patients. g) Representative images of magnetic resonance imaging (MRI) scans before and after induction chemotherapy in responder or non‐responder. NPC tumors are indicated by red lines. h) Bar plot representing the percentage of cases with circWDR37 high or low expression in clinical NPC patient samples from responders (R, *n* = 56) versus non‐responders (NR, *n* = 7). Kaplan–Meier analysis of i) disease‐free survival and j) distant metastasis‐free survival in induction chemotherapy responders with low circWDR37 expression (*n* = 40) and high circWDR37 expression (*n* = 16) groups. k) Proposed model of the mechanism underlying the function of circWDR37 in NPC cell metastasis and senescence resistance. (Data were analyzed by (a–c, i, j) log‐rank test, (d–f) cox proportional hazards regression model, and (h) Chi‐square test). *p*‐value < 0.05 indicates statistical significance.

In light of these findings, we next aimed to validate the clinical potential of circWDR37 expression in 140 NPC tissues using RT‐qPCR. Patients were classified into groups with high (*n* = 101) or low circWDR37 (*n* = 39) expression based on the X‐tile analysis. As shown in Table S3, Supporting Information, circWDR37 expression was significantly and positively correlated with the risks of death and metastasis in NPC patients (*p* < 0.05). Survival analysis demonstrated that patients with high circWDR37 expression showed significantly worse overall survival, disease‐free survival (DFS), and distant metastasis‐free survival (DMFS) than those with low circWDR37 expression (*p* < 0.05, **Figure** **8**a–c). Further analysis revealed that circWDR37 expression level, N stage and pre‐treatment plasma EBV DNA were independent prognostic factors for NPC (*p* < 0.05, Figure 8d–f, Table S4, Supporting Information). These findings show that high circWDR37 expression indicates an unfavorable prognosis and is associated with distant metastasis in NPC patients.

Taking advantage of the above cohort in which 63 patients received gemcitabine or cisplatin induction chemotherapy before radiotherapy, we evaluated the association between circWDR37 expression and patients’ response to cisplatin or gemcitabine induction chemotherapy. Patients underwent magnetic resonance imaging before and after induction chemotherapy (Figure 8g). Low levels of circWDR37 were found in the majority of patient responders (40 out of 56) (Figure 8h), who showed favorable DFS and DMFS compared with those with high levels of circWDR37 (*p* < 0.05, Figure 8i,j), indicating that patients with low circWDR37 expression gain better response and survival benefit from cisplatin or gemcitabine induction chemotherapy. Taken together, these results are consistent with the experimental data showing an association between low circWDR37 expression with the response to cisplatin or gemcitabine chemotherapy and the decreased risks of distant metastasis in NPC patients.

## 3 Discussion

Effective clinical management for preventing and curing metastatic NPC is challenging. Thus, understanding the regulatory mechanisms that drive metastasis is vital for developing more effective therapies for NPC patients. Recent studies have raised the possibility that senescence contributes to tumor progression, recurrence, and metastasis.^[^ ^9^, ^11^, ^47^ ^]^ Initially, senescence limits cell growth due to cell cycle arrest,^[^ ^12^ ^]^ which makes senescence induction a promising anticancer strategy. However, senescence can construct a tumor‐promoting microenvironment through the deleterious SASP.^[^ ^11^ ^]^ Tumor cell senescence can be induced by traditional chemotherapy drugs, including cisplatin and gemcitabine,^[^ ^30^ ^]^ the main treatment regimen for NPC.^[^ ^3^, ^48^, ^49^ ^]^ Moreover, chemotherapy‐induced SASP can facilitate tumor relapse and metastasis.^[^ ^9^, ^11^ ^]^ Therefore, it is important to inhibit the deleterious SASP of senescent tumor cells. Here, we identified that NPC patients developed metastases after radical treatment were characterized with senescence‐ and SASP‐associated phenotypes. Mechanistically, circWDR37 activated PKR to stimulate NF‐*κ*B signaling and facilitate SASP component gene transcription. These findings uncover a novel mechanism in regulating senescence promoted metastasis (Figure 8k).

Here, we identified circWDR37 as a key regulator of chemotherapy‐induced senescence to promote NPC cell metastasis in vitro and in vivo. Recently, therapeutic approaches to target RNAs are being actively pursued due to the endogenous cell machinery and easier designation.^[^ ^50^, ^51^ ^]^ Great effort has been made toward the clinical application of RNA‐based therapeutics, with several approved by the US Food and Drug Administration (FDA), such as siRNAs (e.g., givosiran) that directly interfere with RNA targets.^[^ ^52^ ^]^ Our study found that siRNAs targeting circWDR37 could suppress NPC cell metastasis and chemotherapy‐induced senescence, suggesting si‐circWDR37 as a potential therapeutic strategy for NPC. Due to the covalently closed loop structure, circRNAs are resistant to RNase and stable inside cells and in extracellular fluids, including blood and plasma.^[^ ^53^ ^]^ Accumulating evidence demonstrated that circRNA expression in tissues, plasma or serum could predict prognosis and early tumor detection,^[^ ^54^, ^55^, ^56^ ^]^ suggesting their potential as biomarkers. Here, we demonstrated that patients with low circWDR37 expression gained distant metastasis‐free survival and benefit from cisplatin or gemcitabine treatment. These data validated our in vitro and in vivo experimental findings, and suggest that circWDR37 expression may serve as a biomarker to stratify patients with distinct metastatic risks and guide precise chemotherapy.

NF‐*κ*B is a transcription factor complex essential for the cellular response to inflammatory stimuli and stress signals.^[^ ^32^ ^]^ Consistent with its critical roles in inflammation, NF‐*κ*B directly regulates the transcription of key proinflammatory SASP subset genes.^[^ ^13^, ^14^ ^]^ Constitutive activation of NF‐*κ*B signaling, which can promote cancer immune evasion and systemic dissemination, has been frequently observed in NPC.^[^ ^57^ ^]^ However, the endogenous molecule sustaining NF‐*κ*B activation is largely unclear yet. In this study, we demonstrated that circWDR37 activated the NF‐*κ*B pathway by initiating the homodimerization and autophosphorylation of PKR. Silencing circWDR37 reduced NF‐*κ*B triggered transcription of *CCND1* and proinflammatory SASP genes, and thereby suppressed NPC cell metastasis, indicating that the blocked expression of SASP component genes will help to inhibit metastasis.

PKR has been implicated in dsRNA‐induced NF‐*κ*B pathway activation upon viral infections and immune response.^[^ ^36^ ^]^ Recent evidence demonstrated that a subset of circRNAs with a unique secondary structure comprising imperfect 16–26 bp of dsRNA tended to bind, inhibiting PKR phosphorylation and activation.^[^ ^58^ ^]^ This inhibitory mechanism ensures immune homeostasis under normal conditions.^[^ ^58^ ^]^ In contrast, long noncoding RNAs with long dsRNA‐like hairpin structures can interact with PKR and trigger its homodimerization and phosphorylation to activate NF‐*κ*B and promote breast cancer.^[^ ^41^ ^]^ In summary, a long dsRNA structure (>33 bp) promotes PKR homodimerization and activation, and short dsRNAs (16–33 bp) exert the opposite functions.^[^ ^58^ ^]^ Interestingly, in our study, circWDR37 bound to PKR and promoting its homodimerization and phosphorylation, suggesting that circWDR37 may form long imperfect RNA duplexes to activate PKR. With the advancements in designing of structural and in vivo stability, RNA‐target small molecules have been developed to specifically target non‐coding RNA and effectively disrupt its interaction with proteins.^[^ ^59^ ^]^ Here, we identified circWDR37 as a key regulator of NPC metastasis and senescence by interacting with PKR protein to promote its dimerization and phosphorylation. Thus, circWDR37 may be a potential target to develop small molecular drugs to overcome metastasis in NPC.

The mechanisms by which PKR activates the NF‐*κ*B signaling remain largely unknown. Previous studies have shown that PKR promotes IKK*β* phosphorylation.^[^ ^44^ ^]^ However, whether the kinase activity of PKR activation is involved in IKK*β* phosphorylation has been debated.^[^ ^42^, ^43^ ^]^ Our results revealed that, independent of its kinase function, PKR bound to and facilitated IKK*β* phosphorylation, consistent with the previous findings showing that inactive PKR mutants can activate the IKK complex.^[^ ^43^ ^]^ These data indicate that PKR may function as an adapter protein for NF‐*κ*B activation. In the classical pathway, the inhibitory I*κ*B protein binds with and masks the nuclear localization signal of p65/p50 dimers, resulting in the retention of p65/p50 dimers in the cytoplasm.^[^ ^60^ ^]^ p65/p50 dimers can be phosphorylated and translocated to the nucleus due to the phosphorylation by the I*κ*B kinase complex and subsequent ubiquitination and degradation of I*κ*B.^[^ ^61^ ^]^ Although it has been reported that PKR cannot directly phosphorylate p65,^[^ ^37^ ^]^ our results revealed that PKR interacted with p65. More importantly, PKR competed with I*κ*B*α* for the binding site in p65, resulting in the release of p65 from I*κ*B*α* inhibition. In this study, our results reveal a novel mechanism by which PKR activates NF‐*κ*B and show that PKR silencing sensitizes NPC cells to chemotherapy‐induced senescence and inhibits their metastasis.

We acknowledge some limitations of our study. First, although the rescue of activated PKR significantly reversed the NF‐*κ*B signaling, senescent, migratory and invasive phenotypes of circWDR37‐knockdowned NPC cells, other pathways may also be involved in modulating NPC cell senescence. A deeper characterization of senescent NPC cells is therefore required to examine whether circWDR37 modifies other senescence‐related pathways, such as oxidative stress, energy metabolism, and immune response. In addition, the regulation of circWDR37 in senescence has been examined in vitro, and gene‐editing cell and mouse models need to be further constructed to monitor the effects of circWDR37 in senescence in vivo. It should also be noted that the clinical value of circWDR37 expression needs to be validated in large‐scale, multi‐center studies.

In summary, our study reveals that circWDR37‐activated PKR promotes chemotherapy‐induced senescent tumor cells to metastasis through NF‐*κ*B triggered SASP component gene transcription in NPC. Given that depletion of circWDR37 sensitizes tumor cells to chemotherapy‐induced senescence, attenuates metastasis and enhances benefit from chemotherapy, circWDR37 may be a potential biomarker to predict chemotherapy response and an attractive therapeutic target in NPC.

## 4 Experimental Section

### Analysis of Microarray and RNA Sequencing Data

Microarray mRNA expression data of locoregionally advanced NPC tissues with (*n* = 24) and without distant metastasis (*n* = 24) (GSE103611), microarray RNA expression, and circRNA sequencing data of the S18 and S26 cell lines (GSE210410 and GSE137543) were accessed via Gene Expression Omnibus (GEO) database.

The R package clusterProfiler (4.2.2) was used to integrate the GSEA analysis of multiple related pathways. The R packages pheatmap (1.0.12) and ggplot (2 3.3.6) were applied to draw the heatmap of the senescence‐ and SASP‐associated signature expression in NPC patients with and without distant metastasis. Unsupervised PCA plot analysis was performed using the R packages FactoMineR (2.4) and factoextra (1.0.7).

### Cell Lines and Cell Culture

The HEK293T cells (ACTT CRL‐3216), obtained from the American Tissue Culture Collection, were maintained in DMEM medium. HONE1 and HK1 NPC cell lines were cultured in RPMI 1640 (Gibco), S18 and S26 were cultured in DMEM. All media contained 10% FBS (Gibco). The NPC cell lines were kindly provided by Prof. Mu‐sheng Zeng (Sun Yat‐Sen University Cancer Center, Guangzhou, China). The cell lines were authenticated, tested for mycoplasma contamination, and grown for no more than 1 month.

### RNA Extraction and RT‐qPCR

TRIzol reagent (Invitrogen) was used to extract the total RNA from the cell lines. The AllPrep DNA/RNA Mini Kit (QIAGEN GmbH, Hilden, Germany) was applied to isolate total RNA from fresh frozen tissues following the manufacturer's instructions. M‐MLV reverse transcriptase and random primers (Promega) were used to perform RNA reverse transcription. The cDNA product was used to perform quantitative RT‐PCR using regents of the Platinum SYBR Green qPCR master mix (Invitrogen) with a LightCycler 480 Instrument (384‐well, Roche). GAPDH was used as the internal control. The primers used to detect the circRNA and mRNA expression are listed in Table S5, Supporting Information.

### Microarray Sequencing Analysis

Total RNA of the S18 and S26 cell lines was extracted, purified, amplified, and labeled with an Affymetrix WT PLUS Reagent Kit (Affymetrix, Santa Clara, CA, US) to obtain biotin labeled cDNA. The GeneChip Human Transcriptome Array 2.0 array was hybridized and washed using a GeneChip Hybridization, Wash and Stain Kit (Affymetrix), and scanned with an Affymetrix GeneChip Scanner 3000 (Affymetrix). Command Console Software (Affymetrix) was applied to summarize the probe cell intensity data and generalize the CEL file data using default settings. Expression Console was used to normalize the raw data.

### Actinomycin D Assays

HONE1, HK1 and S18 cells were treated with 2 µg mL^−1^ Actinomycin D (Sigma) for 0, 4, 8, 12, or 24 h. The total RNA was extracted and analyzed by RT‐qPCR, and the 0 h group was used as the control for normalization.

### Nuclear and Cytoplasmic Extraction

The NE‐PER Nuclear and Cytoplasmic Extraction Reagents (Invitrogen) were applied to extract the nuclear and cytoplasmic RNA or protein. Then, the RNA and protein were subjected to RT‒qPCR analysis and western blotting, respectively. *β*‐actin and U6 were used as references for cytoplasmic and nuclear localized RNA, respectively.

### RNA FISH‐Immunofluorescence Microscopy

To identify the cellular localization of circWDR37, NPC cells were seeded on glass slips covered in 24‐well plates with. After 24 h, the cells were immobilized and permeabilized, and then hybridized with Cy3‐labeled circWDR37 FISH probe (RiboBio) at 37 °C in the dark overnight, and nuclei were stained with Hoechst (Invitrogen). To detect the colocalization of circWDR37 with PKR, HONE1, HK1 and S18 cells were first hybridized with Cy3‐labeled circWDR37 probes and then blocked with the PBST supplemented with 5% FBS. Subsequently, the cells were incubated with the anti‐PKR antibody (1:500 dilution) at 4 °C in the dark overnight and incubated with Alexa Fluor 488‐conjugated secondary antibodies and Hoechst the next day. Images were scanned with a confocal microscope (Carl Zeiss).

### siRNAs, Plasmids, and Lentiviruses

The siRNAs against circWDR37 or PKR were designed by RiboBio and GenePharma, respectively. The shRNAs against circWDR37 were cloned into a PLKO.1‐RFP vector. Full length of circWDR37 and its flanking reverse complementary sequences was cloned into modified Lv003 lentiviral vector. Flag or HA‐tagged PKR, and HA‐tagged PKR truncation mutants (PKR‐dsRBMs and PKR‐KD) were cloned into a pSin‐EF2‐puro vector; HA or Flag‐tagged IKK*β*, HA‐tagged IKK*β* truncation mutants (IKK*β*‐1, IKK*β*‐2,4, IKK*β*‐2,3, IKK*β*‐3,4), HA or Flag‐tagged p65, HA‐tagged p65 truncation mutants (p65‐RHD and p65‐TAD) and Myc‐tagged I*κ*B*α* were cloned into a pcDNA3.1 vector; GST‐tagged IKK*β*, PKR, PKRT^446DT451D^ and eIF2*α* were cloned into a pGEX‐4T‐1 vector. All plasmids were verified by sequencing. Lipofectamine 3000 (Invitrogen) was used to perform cell transfections following the manufacturer's protocols. Lentiviral plasmids and infection procedures were performed as previously described.^[^ ^27^ ^]^ Table S5, Supporting Information, shows the sequences of siRNAs and shRNAs.

### RNA Sequencing and Bioinformatic Analysis

Total RNA from si‐circWDR37‐ or siSCR‐ transfected S18 cell lines was extracted using an A RNeasy Mini kit (QIAGEN). The TruSeq RNA Sample Preparation Kit was used to synthesize paired‐end libraries, followed by sequencing on an Illumina NovaSeq 6000 Sequencing System. Hisat2 was applied to map paired‐end sequence files (fastq) to the GRCh38 genome. Fragments per kilobase of exon per million reads mapped (FPKM) values were calculated to represent gene abundance. StringTie software was further applied to determine the number of every gene fragment, which was then normalized with the TNM algorithm. The analysis of differential gene expression was performed by the R package edgeR. The differential genes were identified by: |fold change| > 2 and *q*‐value < 0.05. The KEGG analysis (http://www.genome.ad.jp/kegg) was performed with the Enrich R package. The GSEA analysis was conducted using the GSEA software (http://www.gsea‐msigdb.org/).

### Transwell Migration and Invasion Assays

NPC cells (3.5 × 10^4^ for the migration assay and 7 × 10^4^ for the invasion assay) were suspended in serum‐free medium and then cultured in the upper Transwell chambers with 8‐µm pores (Corning, NY, USA). For invasion assays, the chambers were coated with Matrigel (BD Biosciences, NJ, USA). The lower chambers were supplemented with medium containing 10% FBS. After 10–16 h of incubation, the migrated or invaded cells crossed the chamber were fixed in methanol, stained for 2 h by hematoxylin and observed with an inverted microscope.

### Colony Formation Assays

HONE1 cells (800 cells) or HK1 cells (2000 cells) were treated with 5 mg mL^−1^ cisplatin or 8 ng mL^−1^ gemcitabine after seeded in 6‐well plates for 12 h, and then cultured for 8–12 days in an incubator. After colony formation, the cells were fixed by methanol and hematoxylin stained.

### Senescence‐Associated *β*‐Galactosidase Staining

HONE1, S18, and HK1 cells were seeded in 12‐well plates followed by transfection. After 24 h, they were exposed to 5 mg mL^−1^ cisplatin or 8 ng mL^−1^ gemcitabine for another 24 h. Subsequently, a *β*‐galactosidase staining kit (Beyotime) was used to fix and stain cells according to the manufacturer's protocol.

### Western Blot Assay

Proteins were extracted, separated via SDS‐PAGE, and transferred to PVDF membranes (Merck Millipore). Then, the membranes were incubated with 5% BSA or nonfat milk to block the nonspecific proteins. Next, the protein bands were incubated with primary antibody at 4 °C overnight and incubated with the HRP‐linked secondary antibodies on the next day. An ECL detection system (Thermo Fisher Scientific) was applied to detect the protein bands of interest. The unedited western blotting gels are shown in the Figure S8, Supporting Information. Antibody information is listed in Table S6, Supporting Information.

### Dual Luciferase Reporter Assay

The pGMR‐TK Renilla and NF‐*κ*B luciferase reporter plasmids were purchased from Genomeditech. Cells were cotransfected with pGMR‐TK Renilla, a NF‐*κ*B luciferase reporter plasmid and circWDR37‐siRNA or siSCR control after seeding in 24‐well plates for 12 h. After transfection for 36–48 h, the Dual Luciferase Assay Kit (Promega) was used to analyze the relative luciferase activity following the manufacturer's protocol, which was measured with a GloMax 96 Microplate Luminometer (Promega).

### RNA Pull‐Down Assays

Cells washed with ice‐cold PBS were first lysed with coIP buffer (Thermo Scientific). Then, the washed streptavidin‐coated magnetic beads (Invitrogen) were incubated with biotinylated in vitro‐transcribed circWDR37 or the corresponding complementary biotinylated RNA for 60 min. Subsequently, the RNA‐beads mixture was incubated with the prepared protein lysis buffer at 4 °C overnight. The next day, wash buffer was used to wash the beads for ten times. Finally, the collected proteins were used for mass spectrometry or western blot analysis.

### RNA Immunoprecipitation (RIP)

Magna RIP kit (Millipore) was used to perform RIP assays. Briefly, cell lysates, magnetic beads and antibodies (anti‐PKR or IgG) were mixed together on a rotator at 4 °C overnight. Then, the immunoprecipitated RNA and protein purified from the beads were subjected to RT‐qPCR or western blotting.

### Coimmunoprecipitation

Cells were lysed in coIP buffer followed by centrifugation at 12 000 × *g* for 15 min. Subsequently, the corresponding antibodies were added to the supernatant and incubated at 4 °C overnight. The next day, the supernatant and antibody mixture were further incubated with beads at room temperature for 1 h. Then the beads were washed ten times and resuspended in SDS sample buffer to boil for 10 min at 100 °C. The immunoprecipitates were subjected to western blot analysis.

### Purification of Recombinant Proteins

GST tagged eIF2*α*, PKR, PKR^T446DT451D^, and IKK*β* were cloned into a pGEX‐4T‐1 vector and expressed in Rosetta chemically competent cells (TIANGEN). Affinity chromatography was applied to purify the recombinant proteins with glutathione Sepharose 4B (Cytiva). The purified proteins were dialyzed in NETN buffer and stored at −80 °C. The NETN buffer comprised 20 mm Tris–HCl at pH 8.0, 100 mm NaCl, 1 mm EDTA, and 0.5% NP‐40.

### GST Pull‐Down Assay

GST‐PKR and GST‐PKR^T446DT451D^ were purified in vitro. Twenty micrograms of GST‐PKR or GST‐PKR^T446DT451D^ was incubated with 20 µL glutathione Sepharose 4B at 4 °C overnight. The next day, the beads were washed with NETN buffer six times. HA‐p65, HA‐IKK*β*, or myc‐I*κ*B*α* were transfected into 293T cells, and 48 h later, whole cells were lysed. The cell lysates were then incubated with purified protein‐conjugated beads at 4 °C overnight while rotating. The beads were washed with NETN buffer six times and then subjected to western blot analysis to detect the indicated proteins.

### In Vitro Kinase Assays

GST‐PKR, GST‐eIF2*α*, and GST‐IKK*β* were purified in vitro. For the kinase reaction in vitro, purified GST‐PKR was incubated with purified substrates (GST‐eIF2*α* or GST‐IKK*β*), 10 mm adenosine 5′‐triphosphate, protease inhibitor cocktail, and kinase assay buffer at 37 °C for 30 min. SDS loading buffer was added to stop the kinase reactions, and then, samples were boiled at 100 °C for 5 min in preparation for western blot analysis. The 10× kinase assay buffer comprised 200 mm Tris‐HCl at pH = 7.5, 500 mm KCl, and 100 mm MgCl_2_.

### In Vivo Nude Mouse Models

All animal experiments were performed on 4‐ to 5‐week‐old female BALB/c nude mice, which were purchased from the Beijing Vital River Laboratory Animal Technology Company Co., Ltd. A total of 2 × 10^5^ S18 cells with or without stable circWDR37 knockdown, were injected into the mice footpads (*n* = 7 per group) to establish inguinal lymph node metastasis models. For lymph node metastasis models treated with cisplatin and gemcitabine, the mice in two group were intraperitoneally injected with cisplatin (5 mg kg^−1^) and gemcitabine (50 mg kg^−1^) every 3 days after tumor establishment (a total of 6 times of cisplatin and gemcitabine treatment). After 28 days of tumor injection, the tumors in the footpad and inguinal lymph nodes were excised for H&E staining and IHC staining with an anti‐pan‐cytokeratin antibody (Thermo Fisher Scientific). To construct the lung metastasis model, 1 × 10^6^ S18 cells were injected with or without stable circWDR37 knockdown into the tail vein of the mice (*n* = 5 per group). After 32 days, the lung tissue samples dissected from sacrificed mice were used to conduct H&E staining. All animal experiments were performed following the guidelines of the Care and Use of Laboratory Animals of the National Institutes of Health, and acquired approval from the Institutional Animal Care and Use Ethics Committee of the Sun Yat‐sen University Cancer Center (L025501202107053).

### Clinical Specimens

A total of 140 fresh frozen NPC samples was collected from Sun Yat‐Sen University Cancer Center (SYSUCC) between July 2010 and December 2016. Patients received no antitumor treatment before fresh frozen NPC sample biopsy. The TNM stage was determined following the 8th edition of the American Joint Committee on Cancer staging system. In addition, all patients in the study received intensity modulated radiotherapy (IMRT) and platinum‐based chemotherapy. Of these patients, 63 patients received cisplatin or gemcitabine‐containing induction chemotherapy and underwent MRI evaluation before and after induction chemotherapy. This study was performed according to the Declaration of Helsinki and has been approved by the Institutional Ethical Review Boards of the Sun Yat‐sen University Cancer Center (G2021‐018‐01). Table S3, Supporting Information, shows the detailed clinicopathological characteristics of these patients.

### Statistical Analysis

The results were obtained independently in at least 3 experiments and are shown as the mean ± standard deviation (SD). X‐tile software (version 3.6.1; Yale University, New Haven, CT, USA) was used to determine the optimal cutoff value to classify patients into groups with high or low circWDR37 expression. The differences between two groups were analyzed by unpaired two‐tailed Student's *t* test or *χ* ^2^ test. One‐way ANOVA or two‐way ANOVA was performed to compare multiple groups. The OS, DFS, and DMFS were analyzed following the Kaplan‒Meier method, and a log‐rank test was applied to calculate differences between groups. Univariate and multivariate Cox proportional hazards models were used to analyze prognostic factors. All statistical analyses were performed with SPSS 22.0 software (IBM, Armonk, NY, USA). A *p*‐value < 0.05 indicated statistical significance. N.S. indicated no significance.

## Conflict of Interest

The authors declare no conflict of interest.

## Author Contributions

Q.L., Y.‐H.Z., C.X., Y.‐L.L., and Y.Z. contributed equally to this work. Y.‐Q.L., L.C., J.M., and Q.L. conceived the study; Y.‐Q.L., Q.L., Y.‐H.Z., C.X., Y.‐L.L., Y.Z., Q.‐M.H., J.‐Y.L., K.‐L.C., H.Q., and N.L. designed and performed the experiments; Y.‐Q.L., J.M., L.C., Q.L., C.X., Y.‐H.Z., Y.‐L.L., and Y.Z. acquired and analyzed data; Y.‐Q.L., L.C., J.M., and Q.L. wrote the manuscript.

## Supporting information

Supporting Information

## Data Availability

Microarray mRNA expression data of locoregionally advanced NPC tissues with (n=24) and without distant metastasis (n=24) were accessed via GEO database (GSE103611, https://www.ncbi.nlm.nih.gov/geo/). The microarray RNA expression and circRNA sequencing data of the S18 and S26 cell lines can be accessed via GEO (GSE210410 and GSE137543, respectively). The raw and processed RNA sequencing data of the S18 cells transfected with si‐circWDR37 or a scramble control were deposited in the GEO (GSE207871). The raw data related to this article are deposited at the Research Data Deposit public platform (http://www.researchdata.org.cn; RDDB2023378121).
