# Supplementary material for: Chemotherapy‐Induced Senescence Reprogramming Promotes Nasopharyngeal Carcinoma Metastasis by circRNA‐Mediated PKR Activation
Source: Adv Sci (Weinh). 2023 Jan 22;10(8):2205668. doi: 10.1002/advs.202205668 (PMC10015868; doi:10.1002/advs.202205668)
Supplement: Supplementary file 1 — Supporting Information [file ADVS-10-2205668-s001.pdf]

ADVANCED SCIENCE
Open Access

Supporting Information

for *Adv. Sci.*, DOI 10.1002/advs.202205668

Chemotherapy-Induced Senescence Reprogramming Promotes Nasopharyngeal Carcinoma Metastasis by circRNA-Mediated PKR Activation

Qian Li, Yu-Heng Zhao, Cheng Xu, Ye-Lin Liang, Yin Zhao, Qing-Mei He, Jun-Yan Li, Kai-Lin Chen, Han Qiao, Na Liu, Jun Ma, Lei Chen* and Ying-Qin Li**

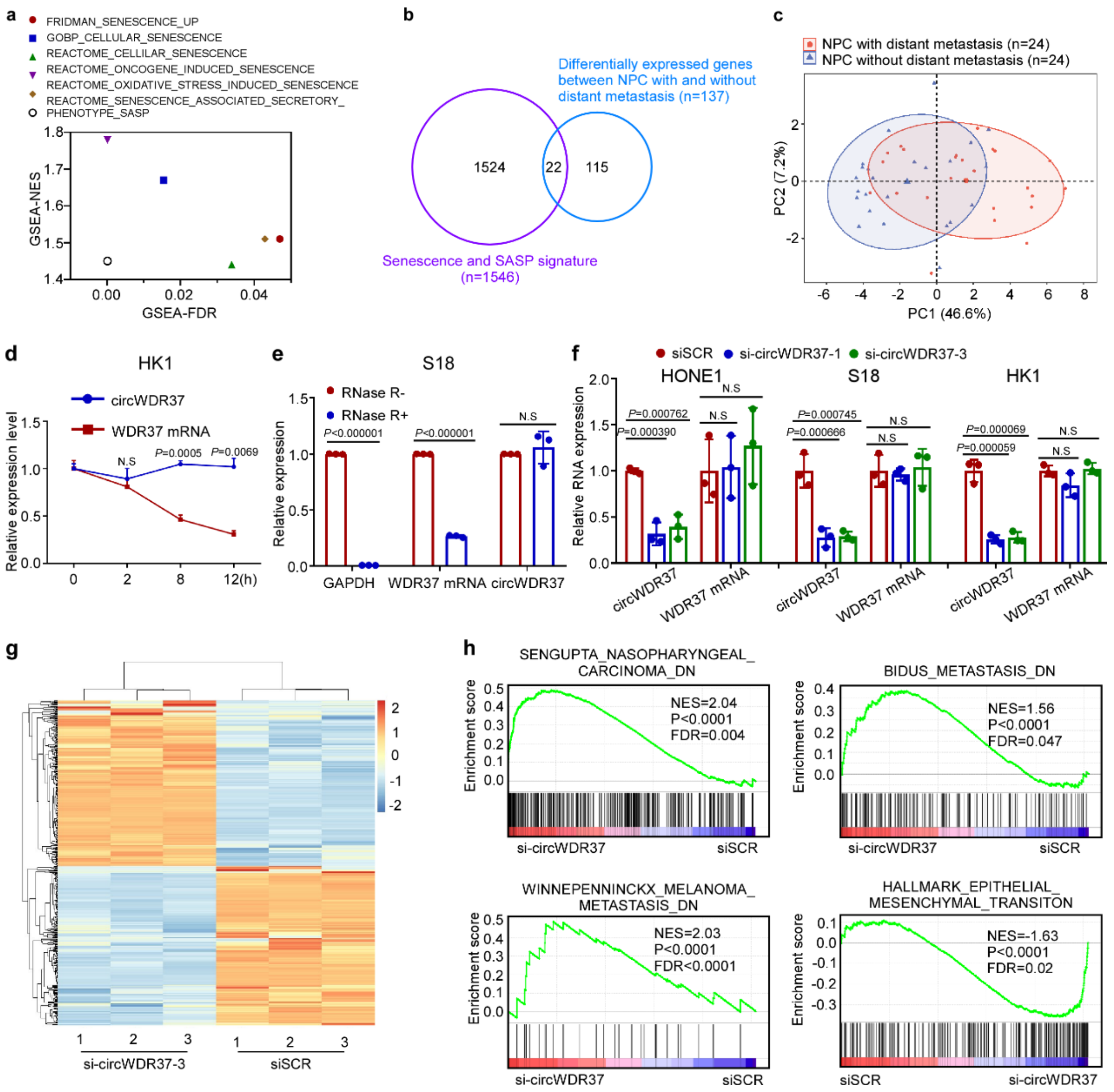

Supplementary figure S1. CircWDR37 is a key regulator in NPC senescence and metastasis. a) GSEA analysis of the microarray sequencing results of 24 paired locoregionally advanced NPC tissues with and without distant metastasis. NES, normalized enrichment score; FDR, false discovery rate. b) Venn diagram showed that 22 of the 137 differentially expressed genes between metastasis and non-metastasis NPC tissues were senescence-associated genes identified in the CellAge database. c) Unsupervised PCA plot analysis of 22 senescence-associated genes via unifrac distance in indicated samples. d) RT-qPCR assay for the expression of circWDR37 and WDR37 mRNA in HK1 cells treated with the transcription inhibitor Actinomycin D (2 μg/ml) at the indicated time points. e) RT-qPCR analysis of GAPDH, WDR37 mRNA and circWDR37 expression after RNase R treatment in S18 cells. f) RT-qPCR results for

circular and linear transcripts of WDR37 in HONE1, S18 and HK1 cells treated with si-circWDR37 (oligonucleotides targeting the back-splice junction) or siSCR (control oligonucleotides with scramble sequence). g) Heat map of the differentially expressed mRNA between three pairs of circWDR37 depleted S18 cells and scramble control (|Fold Change| > 2 and q value < 0.05). h) GSEA analysis of the RNA-seq results of si-circWDR37 and siSCR transfected S18 cells. NES, normalized enrichment score; FDR, false discovery rate. Mean (n = 3) ± s.d. (Data were analyzed by d) two-way ANOVA with Bonferroni's post-hoc test, e) two-tailed Student's t-test) and f) one-way ANOVA with Dunnett's post-hoc test). P value < 0.05 indicates statistical significance. N.S indicates no significant.

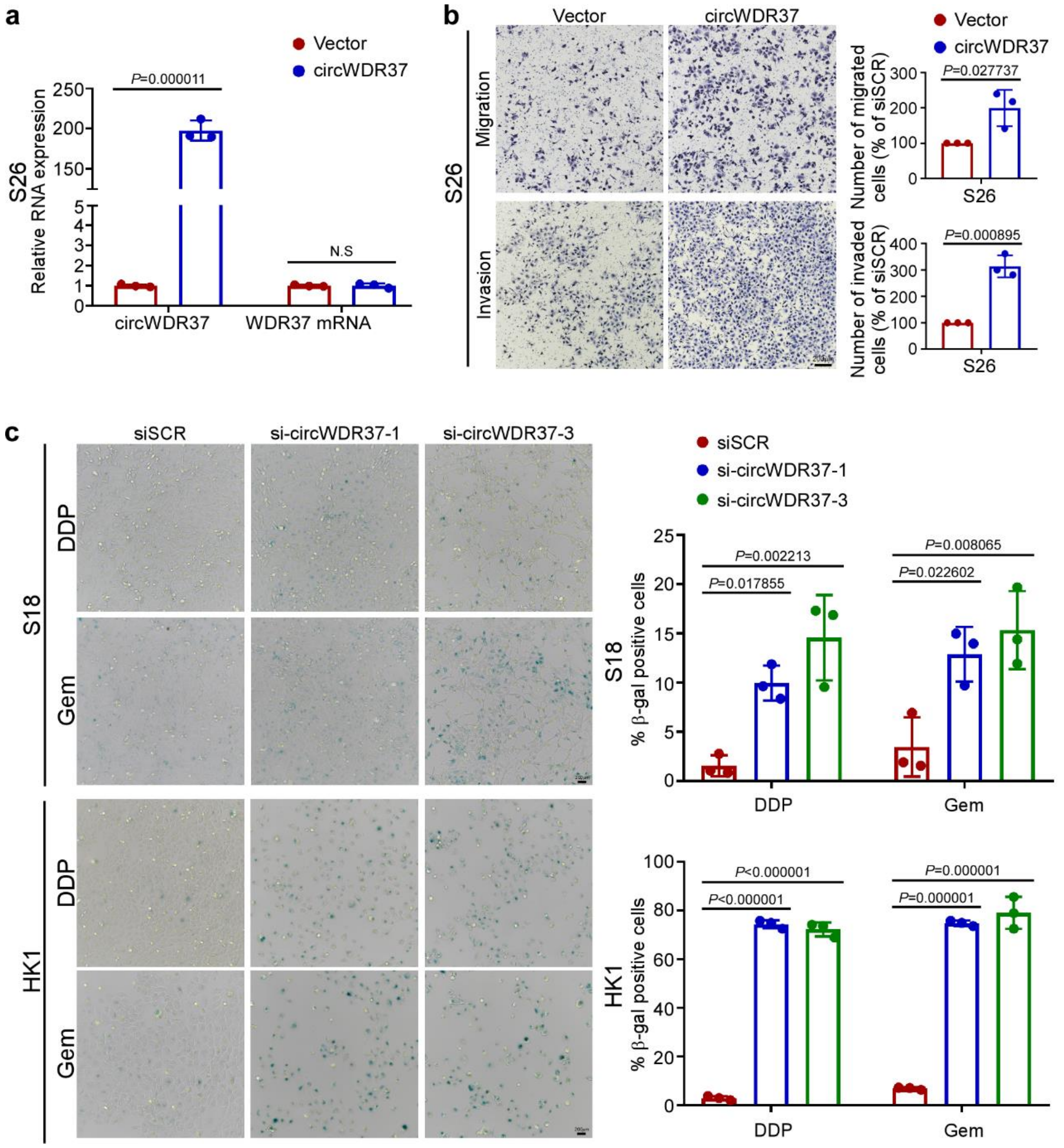

Supplementary figure S2. CircWDR37 deficiency limits chemotherapy-induced senescent NPC cells from promoting metastasis *in vitro*. a) RT-qPCR results for circular and linear transcripts of WDR37 in S26 cells transfected with circWDR37 overexpression plasmid or vector. b) Representative images and quantified results of the transwell migration and invasion assays in S26 cells transfected with circWDR37 overexpression plasmid or vector. c) Representative images and quantified results of SA-*β*-gal staining si-circWDR37 or siSCR transfected S18 and HK1 cells with cisplatin or gemcitabine treated for 24h. Mean (n = 3) ± s.d. (Data were analyzed by a,b) two-tailed Student's t-test and c) one-way ANOVA with Dunnett's post-hoc test). *P* value < 0.05 indicates statistical significance. N.S indicates no significant. Scale bar, 200 μm.

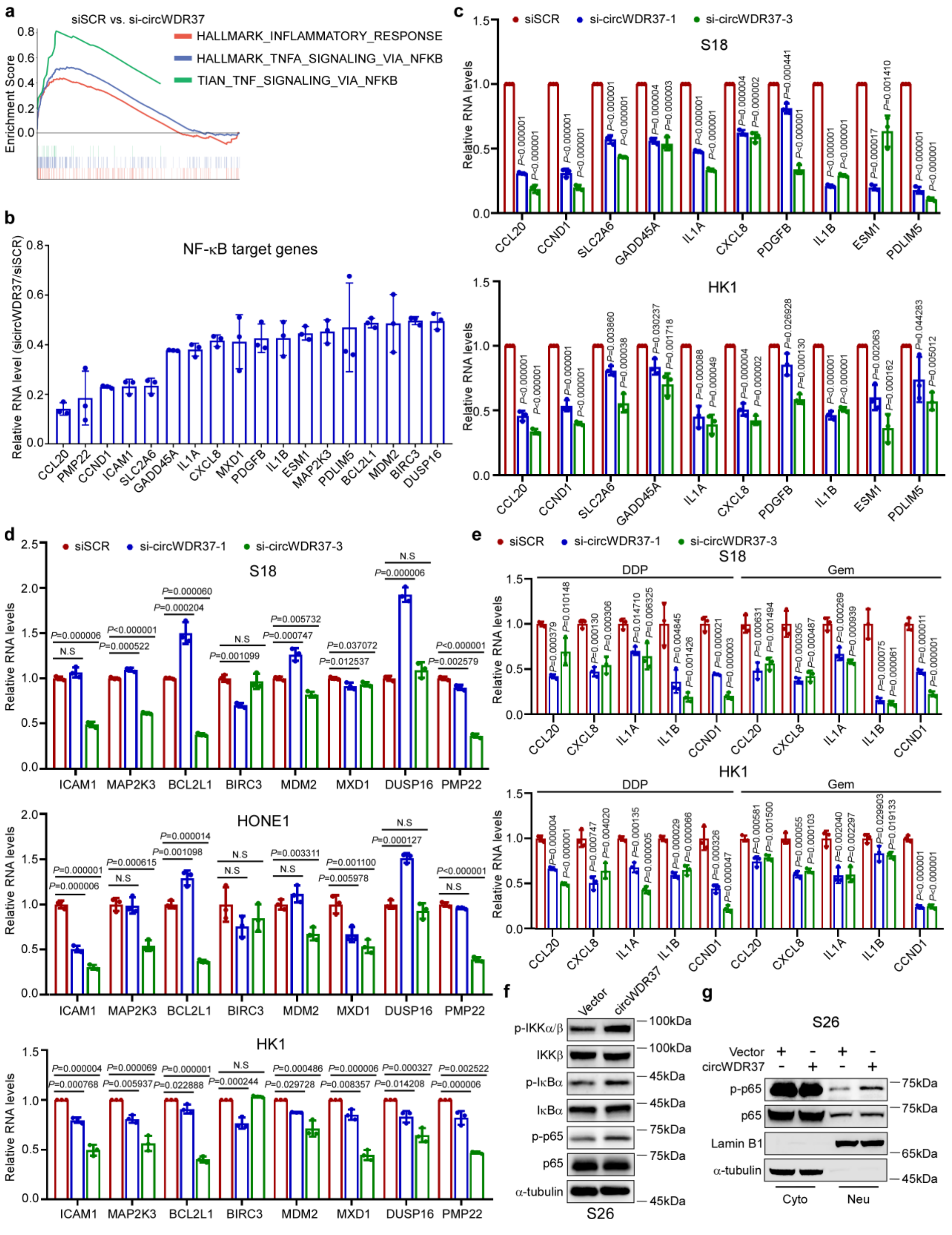

Supplementary figure S3. CircWDR37 stimulates NF-κB-mediated chemotherapy-induced proinflammatory SASP genes transcription. a) GSEA analysis of the RNA-seq results of si-circWDR37 and siSCR transfected S18 cells. b) RNA-seq results of mRNA expression of the indicated NF-κB-targeted genes in circWDR37 knockdown S18 cells. c) RT-qPCR results for the mRNA expression of the indicated NF-κB-targeted genes in S18 and HK1 cells with circWDR37 knockdown. d) RT-qPCR results for the mRNA

expression of the indicated NF-κB-targeted genes, which had a different change in direction in HONE1, S18 and HK1 cells with circWDR37 knockdown. e) RT-qPCR results for the mRNA expression of the SASP component in si-circWDR37 or siSCR transfected S18 and HK1 cells with cisplatin or gemcitabine treated for 24h. f) Western blots analysis of indicated proteins in S26 cells transfected with circWDR37 overexpression plasmid or control plasmid. g) Western blots analysis of the cytoplasmic (cyto) and nuclear (neu) fractions of S26 cells transfected with circWDR37 overexpression plasmid or vector. Lamin B1 and α-tubulin were used as nuclear and cytoplasmic markers, respectively. Mean (n = 3) ± s.d. (c-e) Data were analyzed by one-way ANOVA with Dunnett's post-hoc test). *P* value <0.05 indicates statistical significance. N.S indicates no significant.

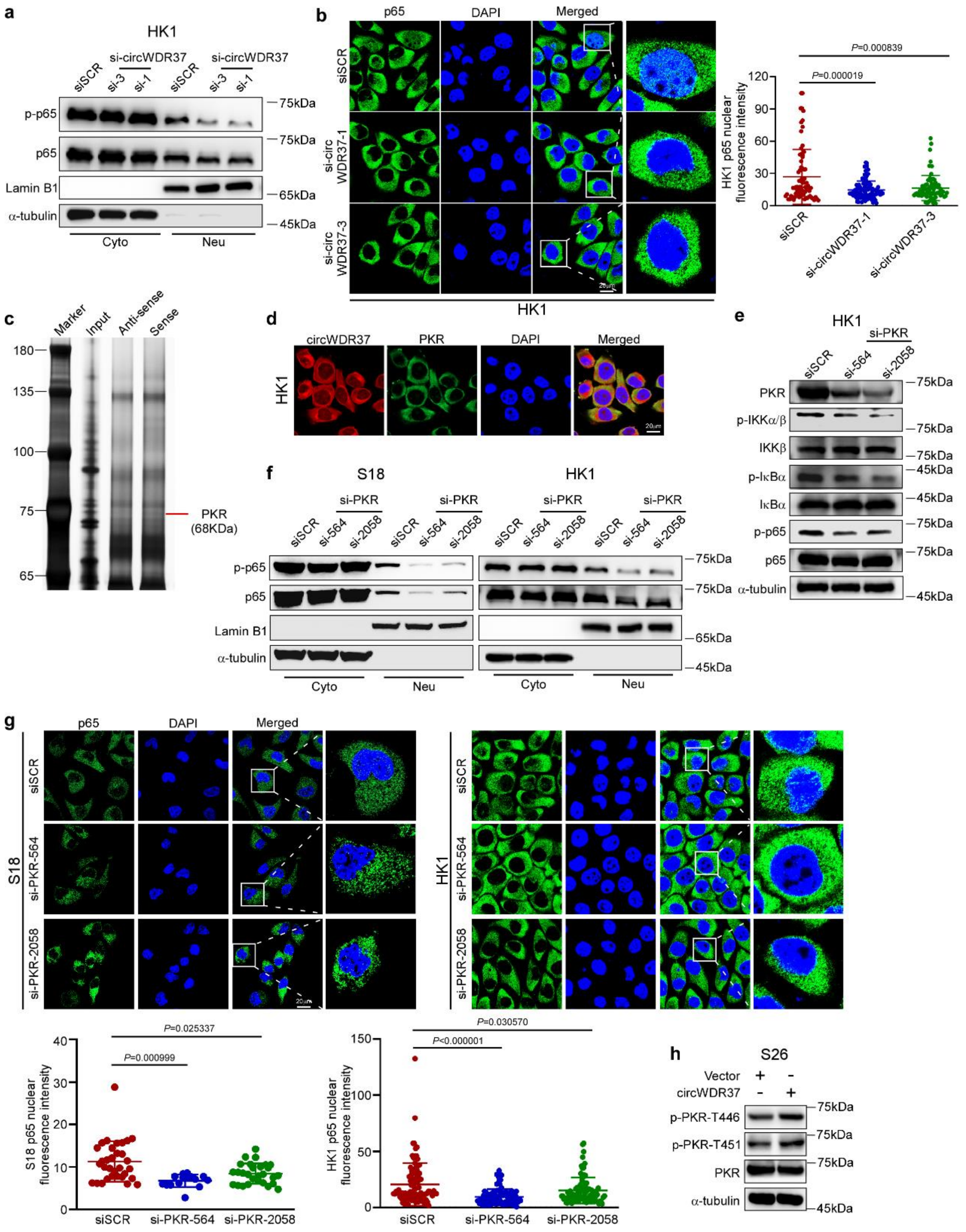

Supplementary figure S4. CircWDR37 induces PKR homodimerization and phosphorylation. a) Western blots analysis of the indicated proteins in cytoplasmic (cyto) and nuclear (neu) fractions of HK1 cells transfected with si-circWDR37 or siSCR. Lamin B1 and α-tubulin were used as nuclear and cytoplasmic markers, respectively. b) Fluorescence microscopy analysis of HK1 cells transfected with si-circWDR37 or siSCR, p65 nuclear fluorescence intensity was determined by ImageJ software. Nuclei were stained with DAPI (blue). Scale bar, 20 μm. c) Biotin-labeled in vitro transcripted

circWDR37 (sense) and corresponding complementary biotinylated RNA (antisense) were incubated with S18 total cell lysates for RNA pull down assays. The silver staining showed the sense-specific band at about 68kDa (red arrow). d) Co-localization of circWDR37 (red) with PKR proteins (green), respectively, in HK1 cells. Scale bar, 20 μm. e) Western blots analysis of the indicated proteins in HK1 cells treated with si-PKR or siSCR. f) Western blots analysis of the indicated proteins in cytoplasmic (cyto) and nuclear (neu) fractions of S18 and HK1 cells transfected with si-PKR or siSCR. Lamin B1 and α-tubulin were used as nuclear and cytoplasmic markers, respectively. g) Fluorescence microscopy analysis of S18 and HK1 cells transfected with si-PKR or siSCR, p65 nuclear fluorescence intensity was determined by ImageJ software. Nuclei were stained with DAPI (blue). Scale bar, 20 μm. h) Western blots analysis of total PKR and phosphorylated PKR (p-PKR-T446, p-PKR-T451) in S26 cells transfected with circWDR37 overexpression plasmid or vector. (b,g) Data were analyzed by one-way ANOVA with Scheffe’s post-hoc test). *P* value <0.05 indicates statistical significance.

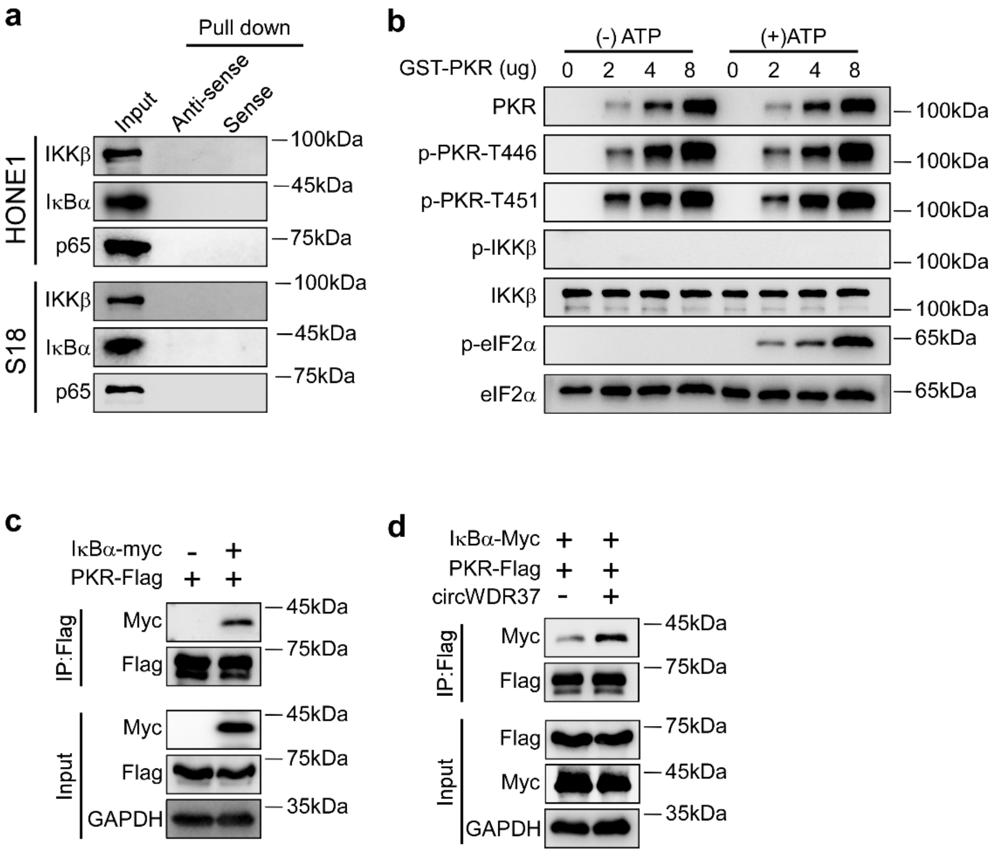

Supplementary figure S5. CircWDR37 promotes the binding of PKR to IKKβ and facilitates the interaction of PKR with IκBα thus releasing p65 from inhibitory IκBα. a) Biotin-labeled in vitro transcripted circWDR37 (sense) and corresponding complementary biotinylated RNA (antisense) were incubated with HONE1 and S18 total cell lysates for RNA pull down assays, the precipitates and whole-cell lysates were then analyzed by western blots with indicated antibodies. b) PKR kinase activity on eIF2α and IKKβ were determined with an in vitro kinase assay, GST-PKR, GST-eIF2α and was GST-IKKβ were in vitro purified. c) Lysates from HEK293T cells co-transfected with plasmids expressing PKR-Flag, along with IκBα-myc were immunoprecipitated with an anti-Flag antibody, the precipitates and whole-cell lysates were then analysed by western blots with indicated antibodies. d) Lysates from HEK293T cells co-transfected with PKR-Flag and IκBα-myc along with circWDR37 or control plasmid were immunoprecipitated with anti-Flag antibody, the precipitates and whole-cell lysates were then analysed by western blots with indicated antibodies.

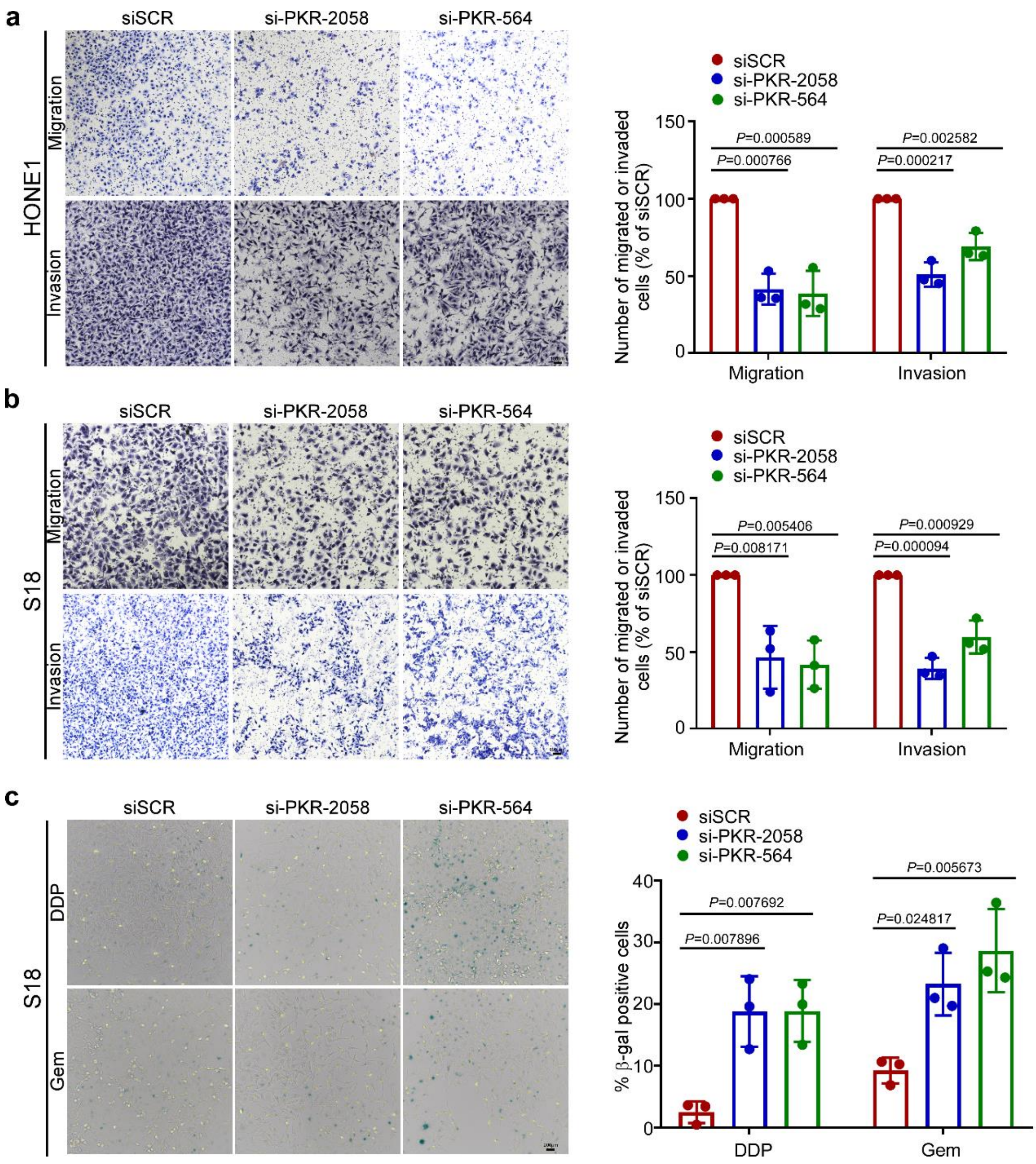

Supplementary figure S6. PKR is a functional mediator of circWDR37. a,b) Representative images and quantified results of the transwell migration and invasion assays in HONE1 (a) and S18 (b) cells transfected with si-PKR or siSCR. Scale bar, 100 μm. c) Representative images and quantified results of SA-*β*-gal staining si-PKR or siSCR transfected S18 cells with cisplatin or gemcitabine treatment. Scale bar, 200 μm. Mean (n = 3) ± s.d. (a-c) Data were analyzed by one-way ANOVA with Dunnett's post-hoc test). *P* value < 0.05 indicates statistical significance. N.S indicates no significant.

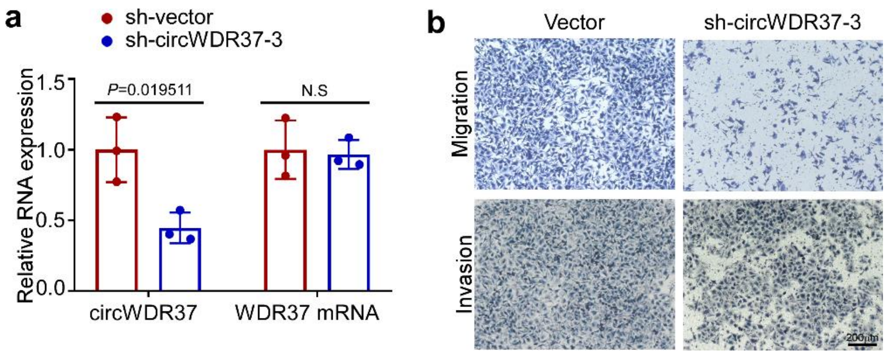

Supplementary figure S7. Migration and invasion abilities of S18 cells with circWDR37 stable knockdown. a) RT-qPCR results for circular and linear transcripts of WDR37 in S18 cells with sh-circWDR37 stable expression or its scramble control. b) Representative images of the transwell migration and invasion assays in S18 cells with sh-circWDR37 stable expression or its scramble control. Scale bar, 200 μm. (a) Data were analyzed by two-tailed Student's t-test). *P* value <0.05 indicates statistical significance. N.S indicates no significant.

Supplementary figure S8. Full unedited gels for all figures (continued).

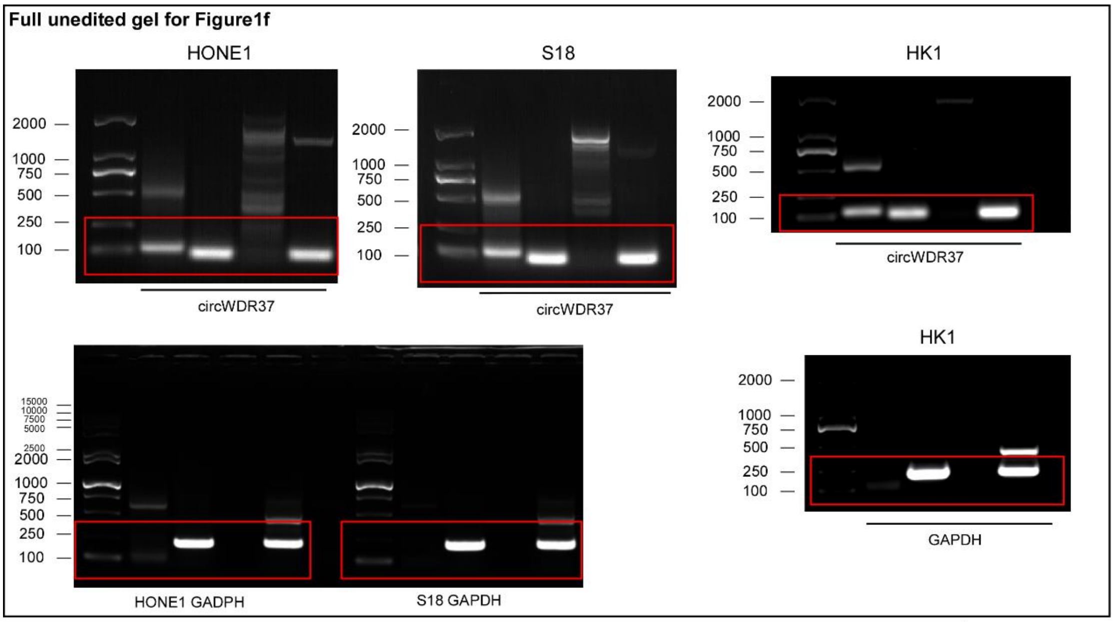

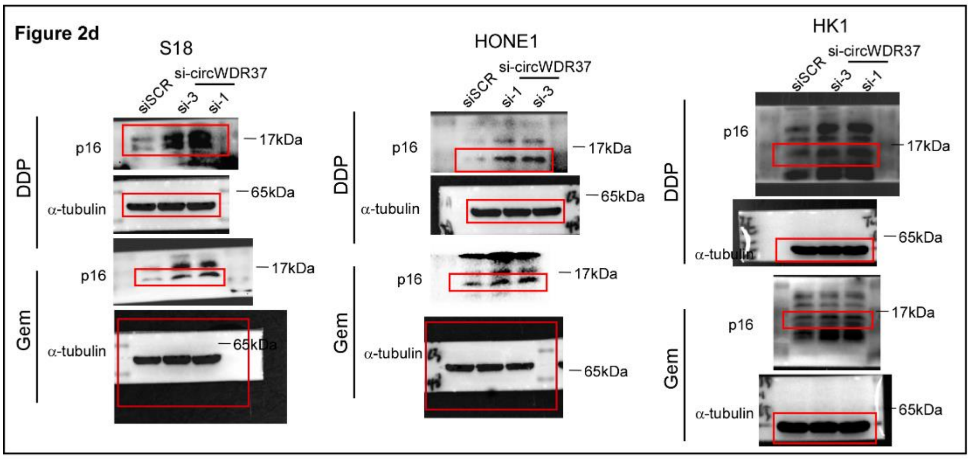

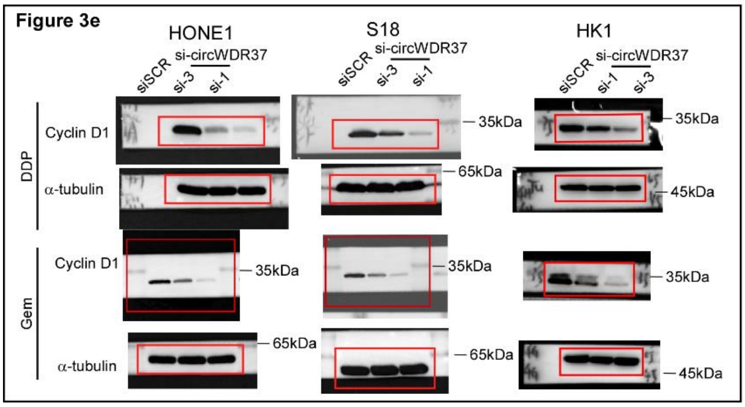

Supplementary figure S8. Full unedited gels for all figures (continued).

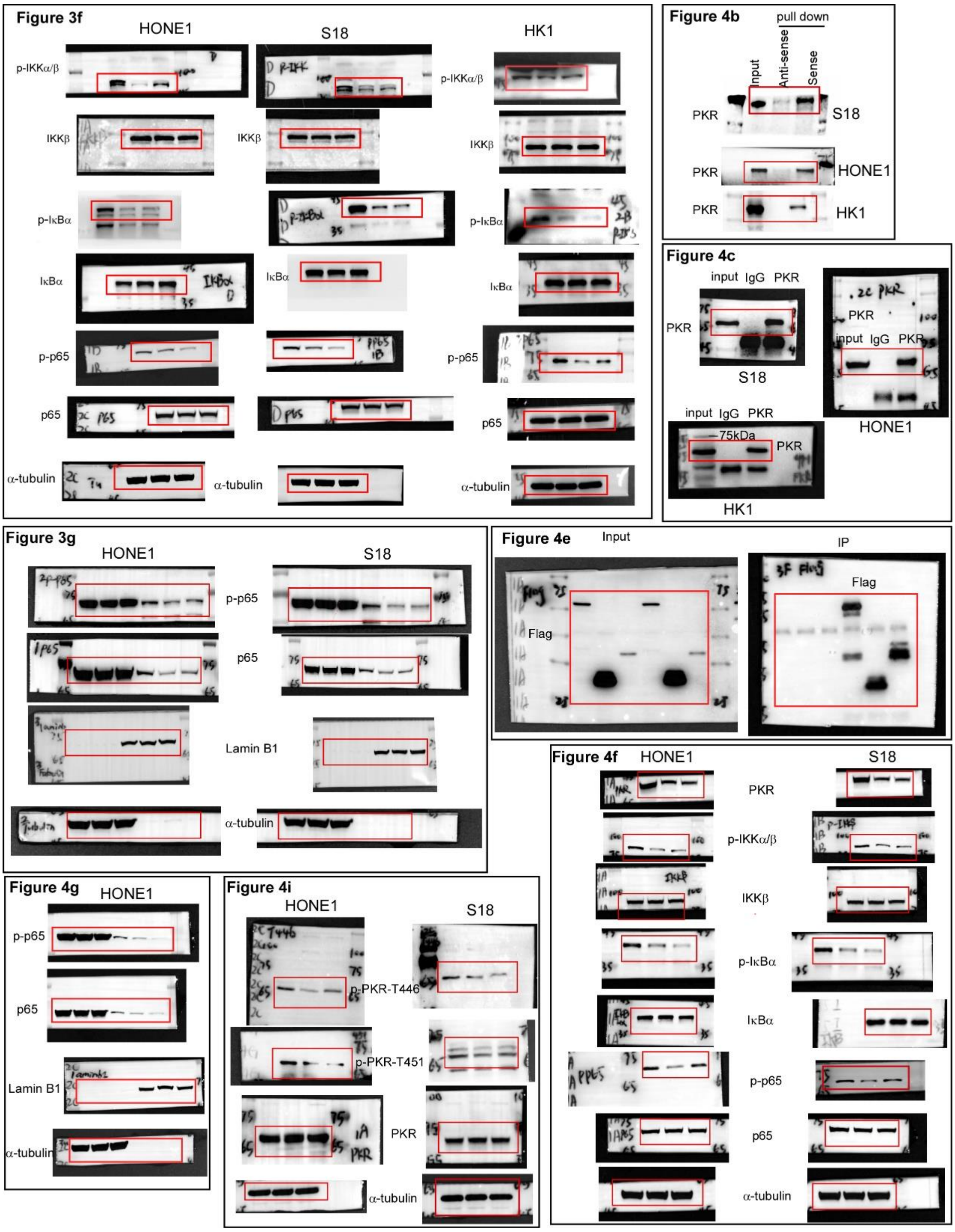

Supplementary figure S8. Full unedited gels for all figures (continued).

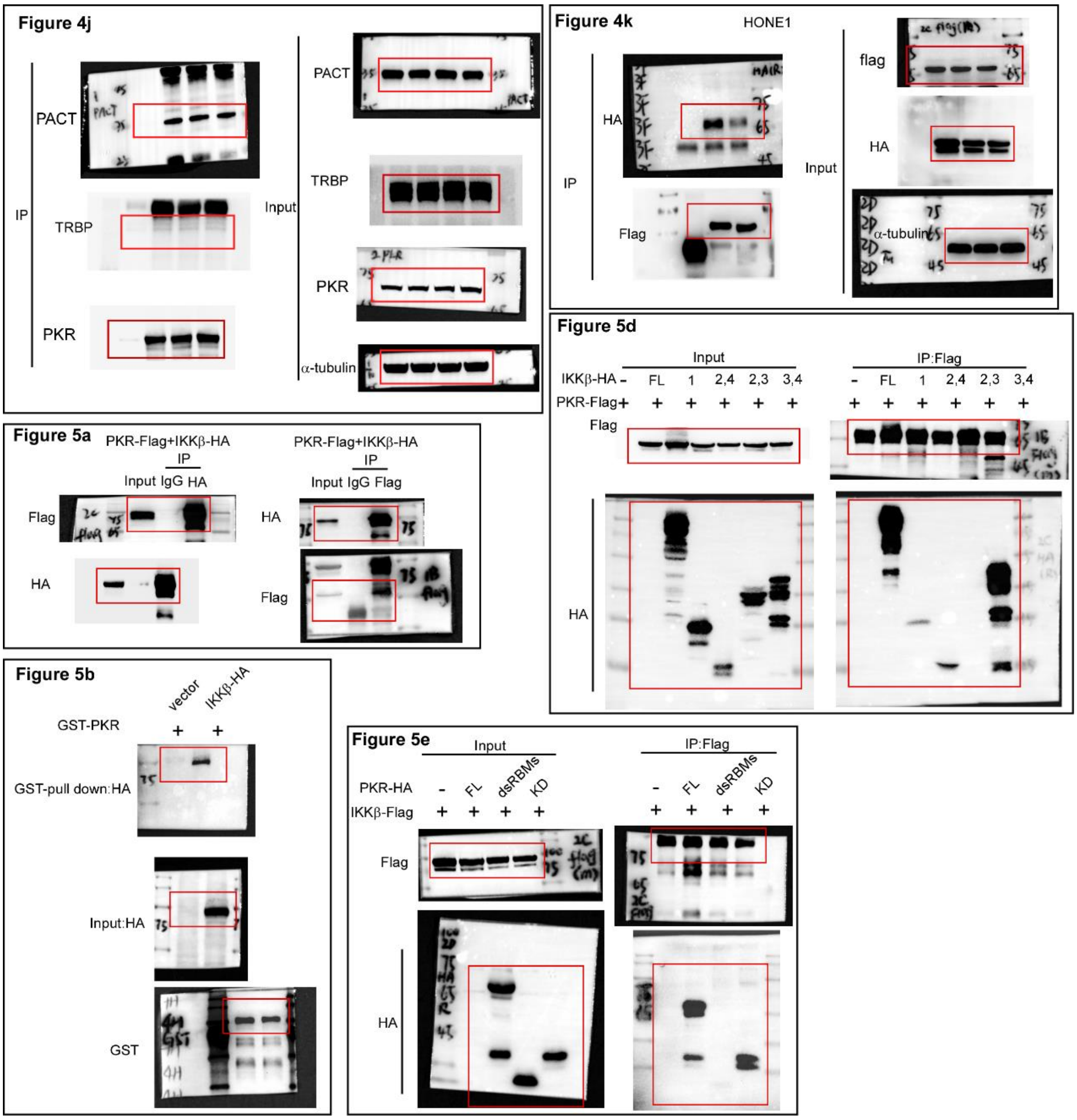

Supplementary figure S8. Full unedited gels for all figures (continued).

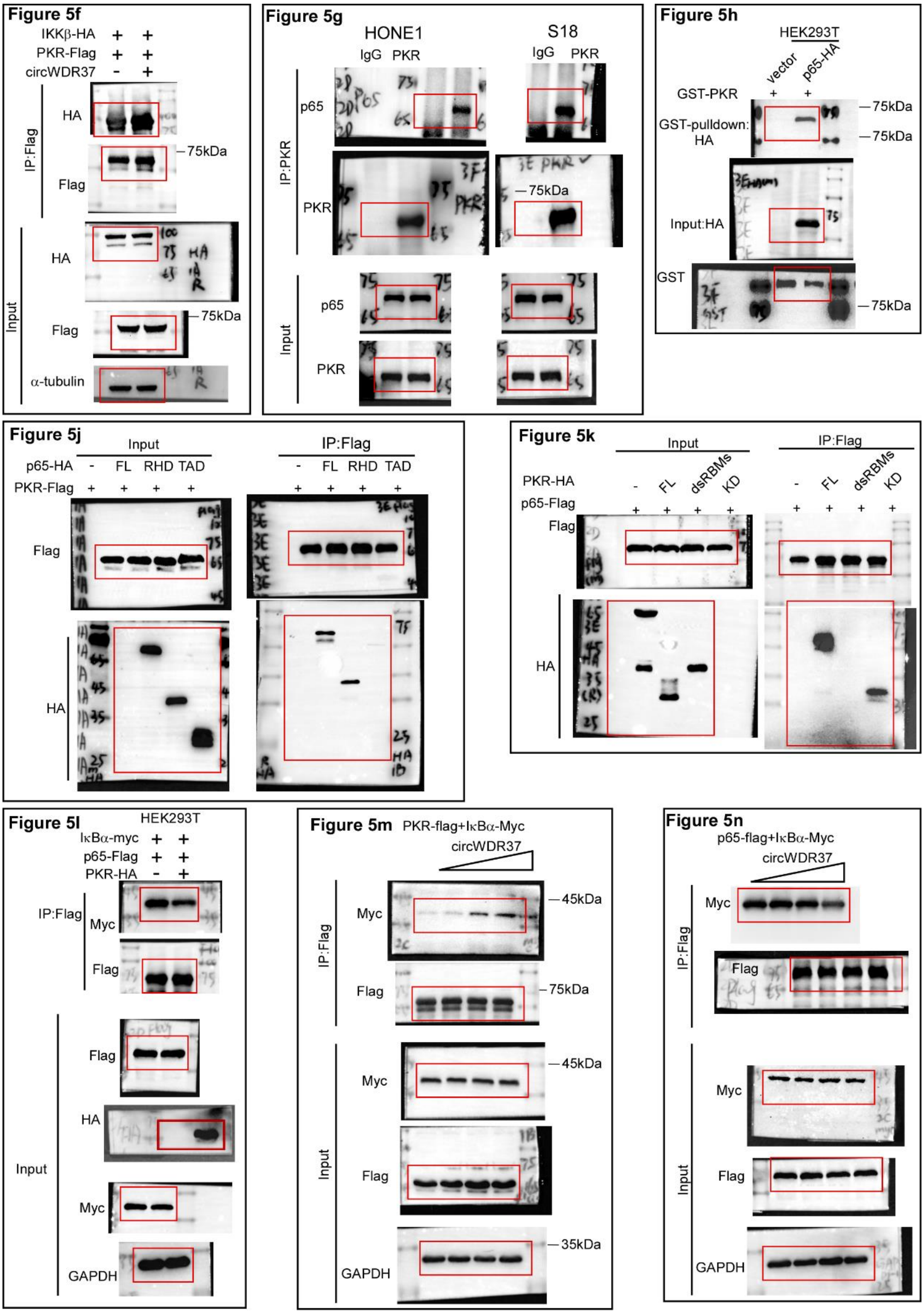

Supplementary figure S8. Full unedited gels for all figures (continued).

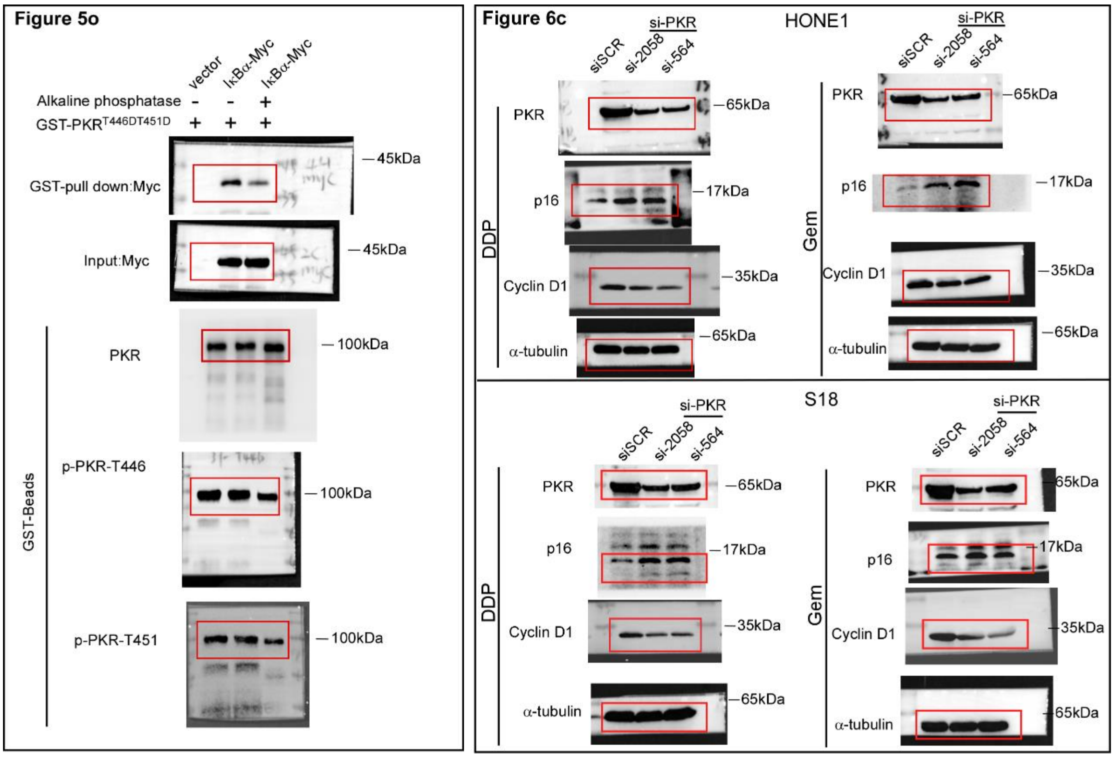

Supplementary figure S8. Full unedited gels for all figures (continued).

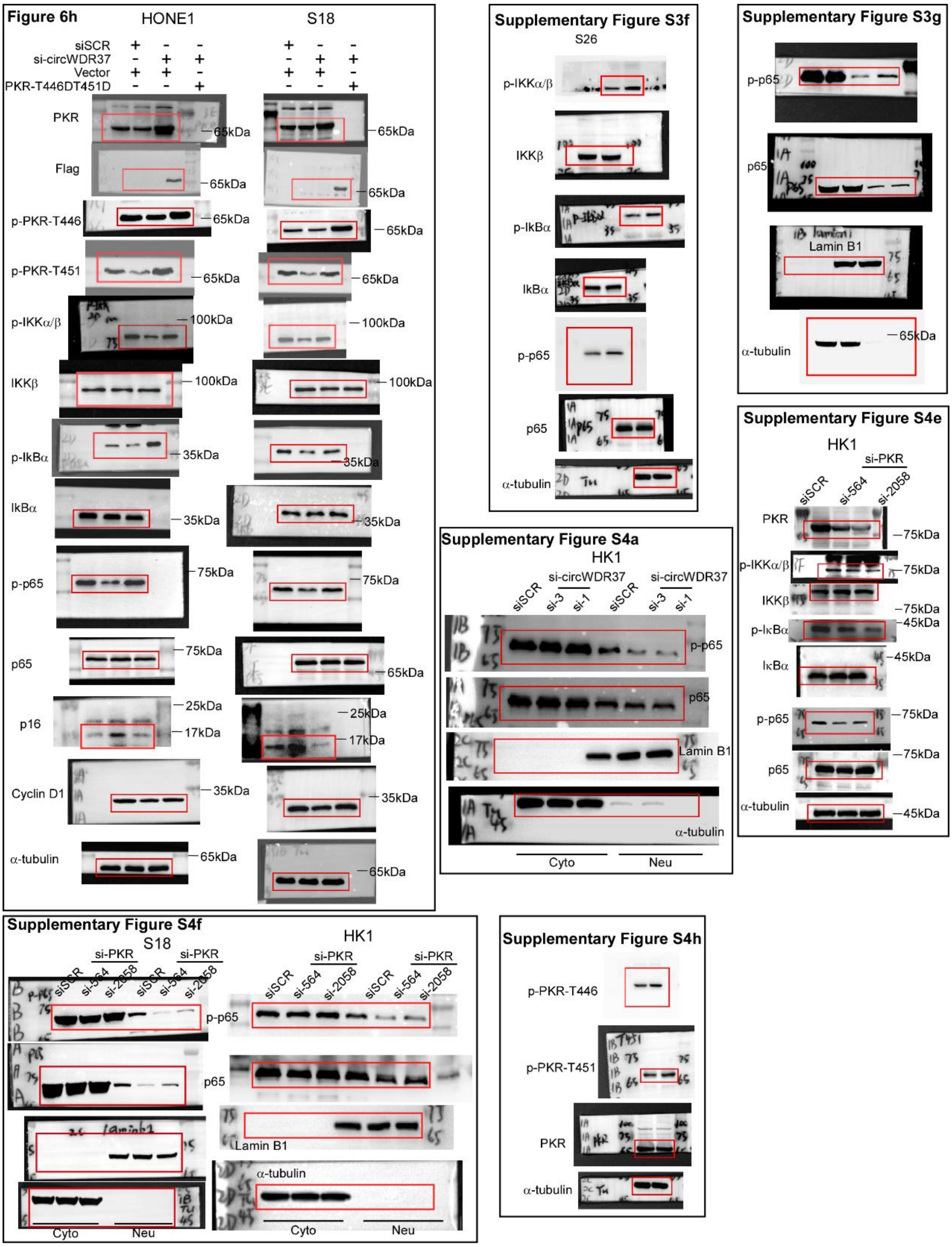

Supplementary figure S8. Full unedited gels for all figures (continued).

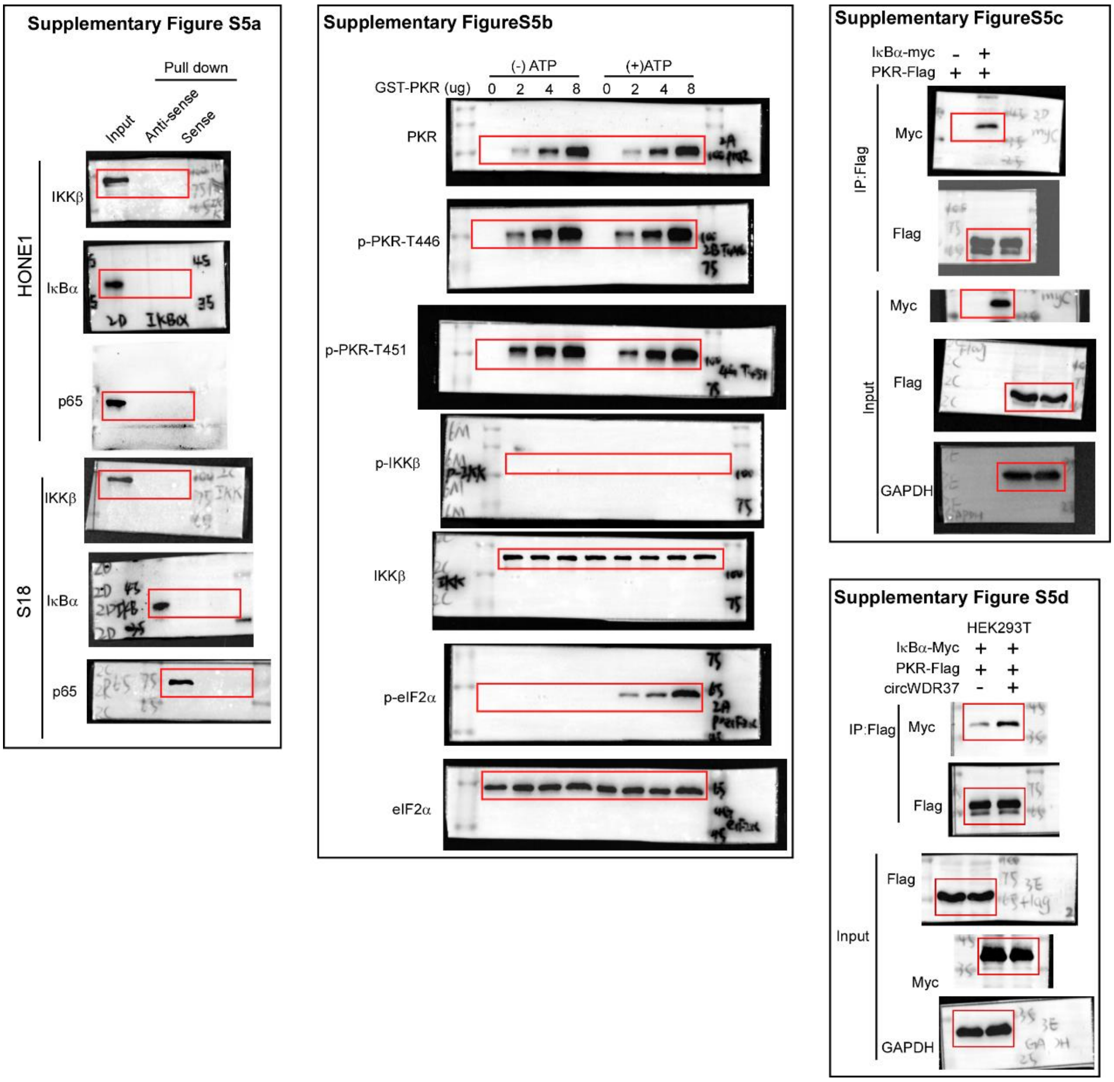

Supplementary Table S1. The CSCD database information of the 40 circRNAs validated via 4 different algorithms

Number	AccID	circBase ID	ratio(circRNA/linear)	algorithm
1	chr10_1142186_1130343_+11843-	hsa_circ_0000206	0.817483225	find_circ,CIRI,circRNA_finder,circexplor
2	chr5_38530768_38523521_-7247-LIFR	hsa_circ_0072309	0.742648174	find_circ,CIRI,circRNA_finder,circexplor
3	chr17_81006661_81006347_-314-	hsa_circ_0000819	0.732376141	find_circ,CIRI,circRNA_finder,circexplor
4	chr8_131414216_131370263_-43953-	hsa_circ_0003691	0.718631125	find_circ,CIRI,circRNA_finder,circexplor
5	chr3_196534785_196533450_+1335-	hsa_circ_0003036	0.71154252	find_circ,CIRI,circRNA_finder,circexplor
6	chr11_122647918_122646927_+991-	hsa_circ_0003391	0.704525435	find_circ,CIRI,circRNA_finder,circexplor
7	chr2_36669878_36623757_+46121-	hsa_circ_0002346	0.695870364	find_circ,CIRI,circRNA_finder,circexplor
8	chr9_134526336_134518626_-7710-	hsa_circ_0089254	0.684453123	find_circ,CIRI,circRNA_finder,circexplor
9	chr12_111856681_111855923_+758-	hsa_circ_0006741	0.680562502	find_circ,CIRI,circRNA_finder,circexplor
10	chr15_71447271_71433788_+13483-	hsa_circ_0005203	0.662019093	find_circ,CIRI,circRNA_finder,circexplor
11	chr1_167944253_167935867_+8386-	hsa_circ_0009109	0.660022996	find_circ,CIRI,circRNA_finder,circexplor
12	chr7_100454798_100454490_+308-	hsa_circ_0007639	0.654949866	find_circ,CIRI,circRNA_finder,circexplor
13	chr7_1938026_1937836_-190-MAD1L1	hsa_circ_0079135	0.644007367	find_circ,CIRI,circRNA_finder,circexplor
14	chr10_49618211_49609655_+8556-	hsa_circ_0002968	0.641119508	find_circ,CIRI,circRNA_finder,circexplor
15	chr4_89400658_89396992_+3666-	hsa_circ_0070421	0.640744622	find_circ,CIRI,circRNA_finder,circexplor
16	chr6_24418806_24416625_+2181-	hsa_circ_0075835	0.634776589	find_circ,CIRI,circRNA_finder,circexplor
17	chr19_46879831_46878861_+970-	hsa_circ_0003356	0.630428141	find_circ,CIRI,circRNA_finder,circexplor
18	chr1_46295253_46290105_+5148-	hsa_circ_0012277	0.629631836	find_circ,CIRI,circRNA_finder,circexplor
19	chr12_29911710_29904599_-7111-	hsa_circ_0025767	0.610061196	find_circ,CIRI,circRNA_finder,circexplor
20	chr20_60922084_60920874_-1210-	hsa_circ_0061099	0.596578291	find_circ,CIRI,circRNA_finder,circexplor

21	chr11_103158334_103151077_+7257-	hsa_circ_0006960	0.593295168	find_circ,CIRI,circRNA_finder,circexplor
22	chr1_98165103_98144651_-20452-	hsa_circ_0004161	0.577576234	find_circ,CIRI,circRNA_finder,circexplor
23	chr13_114277601_114265311_+12290-	hsa_circ_0031038	0.57559983	find_circ,CIRI,circRNA_finder,circexplor
24	chr11_981299_977095_+4204-AP2A2	hsa_circ_0020740	0.571244543	find_circ,CIRI,circRNA_finder,circexplor
25	chr9_135527879_135517391_-10488-	hsa_circ_0089294	0.557402854	find_circ,CIRI,circRNA_finder,circexplor
26	chr2_36623930_36623757_+173-	hsa_circ_0002017	0.556874631	find_circ,CIRI,circRNA_finder,circexplor
27	chr3_30691952_30686239_+5713-	hsa_circ_0005224	0.539544825	find_circ,CIRI,circRNA_finder,circexplor
28	chr6_144860579_144858718_+1861-	hsa_circ_0001647	0.538974393	find_circ,CIRI,circRNA_finder,circexplor
29	chr17_16981390_16979024_+2366-	hsa_circ_0006600	0.526594271	find_circ,CIRI,circRNA_finder,circexplor
30	chr8_26441499_26439485_+2014-	hsa_circ_0005013	0.525701195	find_circ,CIRI,circRNA_finder,circexplor
31	chr10_128860040_128850945_+9095-	hsa_circ_0020460	0.520198432	find_circ,CIRI,circRNA_finder,circexplor
32	chr5_14336836_14270934_+65902-	hsa_circ_0006674	0.509454593	find_circ,CIRI,circRNA_finder,circexplor
33	chr6_34574681_34574332_-349-	hsa_circ_0006757	0.509001025	find_circ,CIRI,circRNA_finder,circexplor
34	chr8_17143936_17137884_+6052-	hsa_circ_0083423	0.508163027	find_circ,CIRI,circRNA_finder,circexplor
35	chr12_56194684_56182859_-11825-	hsa_circ_0007446	0.503978299	find_circ,CIRI,circRNA_finder,circexplor
36	chr1_243388623_243385026_-3597-	hsa_circ_0005749	0.497161563	find_circ,CIRI,circRNA_finder,circexplor
37	chr19_41737203_41736872_+331-AXL	hsa_circ_0002945	0.492995055	find_circ,CIRI,circRNA_finder,circexplor
38	chr3_186505373_186504916_+457-	hsa_circ_0068464	0.470678199	find_circ,CIRI,circRNA_finder,circexplor
39	chr11_9446809_9441958_+4851-IPO7	hsa_circ_0004672	0.413797212	find_circ,CIRI,circRNA_finder,circexplor
40	chr20_34246904_34243124_-3780-	hsa_circ_0060122	0	find_circ,CIRI,circRNA_finder,circexplor

Supplementary Table S2. circWDR37-associated proteins or isoforms identified by mass spectrometry in NPC cells S18

Nu	Accesion	Description	Sc	Ma	Ma	Matc	Sequ	Seque	Co	pi	em
1	sp|Q9Y6M1|IF	Insulin-like growth factor 2 mRNA-binding protein 2 OS=Homo	11	66	2	2	2	2	4.2	8.	0.1
2	sp|P09914|IFI	Interferon-induced protein with tetratricopeptide repeats 1	10	55	7	7	4	4	8.4	6.	0.4
3	sp|Q16629|SR	Serine/arginine-rich splicing factor 7 OS=Homo sapiens	10	27	3	3	2	2	8.8	11	0.2
4	sp|P31942|HN	Heterogeneous nuclear ribonucleoprotein H3 OS=Homo sapiens	97	36	2	2	2	2	6.4	6.	0.1
5	sp|Q7Z2W4|Z	Zinc finger CCCH-type antiviral protein 1 OS=Homo sapiens	88	10	4	4	4	4	5.9	8.	0.1
6	sp|P52209|6PG	6-phosphogluconate dehydrogenase, decarboxylating OS=Homo	71	53	1	1	1	1	2.3	6.	0.0
7	sp|Q07955|SR	Serine/arginine-rich splicing factor 1 OS=Homo sapiens	70	27	3	3	2	2	8.5	10	0.2
8	sp|O75390|CIS	Citrate synthase, mitochondrial OS=Homo sapiens OX=9606	68	51	1	1	1	1	2.4	8.	0.0
9	sp|P19525|E2A	Interferon-induced, double-stranded RNA-activated protein	65	62	3	3	2	2	4.9	8.	0.1
10	sp|P09913|IFI	Interferon-induced protein with tetratricopeptide repeats 2	64	55	5	5	5	5	8.9	6.	0.3
11	sp|O00425|IF2	Insulin-like growth factor 2 mRNA-binding protein 3 OS=Homo	61	64	1	1	1	1	2.2	8.	0.0
12	sp|Q8NBJ5|G	Procollagen galactosyltransferase 1 OS=Homo sapiens OX=9606	60	71	1	1	1	1	2.3	6.	0.0
13	sp|O95573|AC	Long-chain-fatty-acid--CoA ligase 3 OS=Homo sapiens OX=9606	57	81	1	1	1	1	2.1	8.	0.0
14	sp|O75569|PR	Interferon-inducible double-stranded RNA-dependent protein	56	34	1	1	1	1	4.8	8.	0.1
15	sp|Q7Z417|NU	Nuclear fragile X mental retardation-interacting protein 2	55	76	1	1	1	1	1.9	8.	0.0
16	sp|Q9NZI8|IF	Insulin-like growth factor 2 mRNA-binding protein 1 OS=Homo	54	63	2	2	2	2	4.3	9.	0.1
17	sp|Q08211|DH	ATP-dependent RNA helicase A OS=Homo sapiens OX=9606	52	14	2	2	2	2	1.7	6.	0.0
18	sp|P38159|RB	RNA-binding motif protein, X chromosome OS=Homo sapiens	52	42	3	3	2	2	5.1	10	0.1
19	sp|P61221|AB	ATP-binding cassette sub-family E member 1 OS=Homo sapiens	51	68	1	1	1	1	2	8.	0.0
20	sp|P40938|RF	Replication factor C subunit 3 OS=Homo sapiens OX=9606	50	41	1	1	1	1	3.9	8.	0.0
21	sp|Q8TD47|RS	40S ribosomal protein S4, Y isoform 2 OS=Homo sapiens	49	29	2	2	1	1	3.4	10	0.1
22	sp|Q53EU6|G	Glycerol-3-phosphate acyltransferase 3 OS=Homo sapiens	48	49	1	1	1	1	2.8	9.	0.0
23	sp|Q9Y3Y2|C	Chromatin target of PRMT1 protein OS=Homo sapiens	47	26	1	1	1	1	5.2	12	0.1
24	sp|O14879|IFI	Interferon-induced protein with tetratricopeptide repeats 3	47	56	2	2	2	2	3.1	5.	0.1
25	sp|P62841|RS1	40S ribosomal protein S15 OS=Homo sapiens OX=9606	47	17	2	2	1	1	8.3	10	0.4
26	sp|P56589|PE	Peroxisomal biogenesis factor 3 OS=Homo sapiens OX=9606	46	42	1	1	1	1	2.7	8.	0.0
27	sp|Q12849|GR	G-rich sequence factor 1 OS=Homo sapiens OX=9606	45	53	1	1	1	1	2.3	5.	0.0
28	sp|Q15637|SF0	Splicing factor 1 OS=Homo sapiens OX=9606 GN=SF1 PE=1	44	68	1	1	1	1	2	9.	0.0
29	sp|Q99729|RO	Heterogeneous nuclear ribonucleoprotein A/B OS=Homo sapiens	44	36	2	2	2	2	6.9	8.	0.1
30	sp|Q96EP5|DA	DAZ-associated protein 1 OS=Homo sapiens OX=9606	44	43	1	1	1	1	3.7	8.	0.0
31	sp|P39748|FE	Flap endonuclease 1 OS=Homo sapiens OX=9606 GN=FEN1	44	42	2	2	2	2	7.1	8.	0.1
32	sp|Q3ZCQ8|TI	Mitochondrial import inner membrane translocase subunit	41	39	1	1	1	1	4.8	8.	0.0
33	sp|Q9BSR8|YI	Protein YIPF4 OS=Homo sapiens OX=9606 GN=YIPF4 PE=1	40	27	1	1	1	1	3.7	4.	0.1
34	sp|Q15149|PL	Plectin OS=Homo sapiens OX=9606 GN=PLEC PE=1 SV=3	39	53	1	1	1	1	0.4	5.	0.0
35	sp|Q5BKZ1|Z	DBIRD complex subunit ZNF326 OS=Homo sapiens OX=9606	39	65	1	1	1	1	2.2	5.	0.0
36	sp|Q9NXS2|Q	Glutaminyl-peptide cyclotransferase-like protein OS=Homo	38	43	1	1	1	1	2.9	9.	0.0
37	sp|Q13409|DC	Cytoplasmic dynein 1 intermediate chain 2 OS=Homo sapiens	38	71	1	1	1	1	3.3	5.	0.0
38	sp|Q5JWF2|G	Guanine nucleotide-binding protein G(s) subunit alpha isoforms	38	11	1	1	1	1	1.5	4.	0.0
39	sp|O43818|U3I	U3 small nucleolar RNA-interacting protein 2 OS=Homo sapiens	36	52	1	1	1	1	2.1	7.	0.0
40	sp|Q96AE4|FU	Far upstream element-binding protein 1 OS=Homo sapiens	36	67	2	2	2	2	3.6	7.	0.1
41	sp|Q7LGA3|H	Heparan sulfate 2-O-sulfotransferase 1 OS=Homo sapiens	35	42	1	1	1	1	4.8	8.	0.0
42	sp|Q92945|FU	Far upstream element-binding protein 2 OS=Homo sapiens	35	73	1	1	1	1	1.8	6.	0.0
43	sp|Q13325|IFI	Interferon-induced protein with tetratricopeptide repeats 5	35	56	2	2	2	2	3.7	7	0.1
44	sp|P31040|SD	Succinate dehydrogenase [ubiquinone] flavoprotein subunit,	34	73	1	1	1	1	1.7	7.	0.0
45	sp|Q9BU23|L	Lipase maturation factor 2 OS=Homo sapiens OX=9606	34	79	1	1	1	1	3	10	0.0
46	sp|Q9Y3I0|RT	RNA-splicing ligase RtcB homolog OS=Homo sapiens OX=9606	34	55	1	1	1	1	2.2	6.	0.0
47	sp|Q15633|TR	RISC-loading complex subunit TARBP2 OS=Homo sapiens	34	39	1	1	1	1	4.1	6.	0.0
48	sp|Q9H7F4|T1	Transmembrane protein 185B OS=Homo sapiens OX=9606	33	40	1	1	1	1	2.6	7.	0.0
49	sp|O43291|SPI	Kunitz-type protease inhibitor 2 OS=Homo sapiens OX=9606	33	28	1	1	1	1	4.8	8.	0.1
50	sp|O96006|ZB	Zinc finger BED domain-containing protein 1 OS=Homo sapiens	31	78	1	1	1	1	1	5.	0.0
51	sp|Q13347|EIF	Eukaryotic translation initiation factor 3 subunit I OS=Homo	31	36	1	1	1	1	3.7	5.	0.0
52	sp|P37108|SRP	Signal recognition particle 14 kDa protein OS=Homo sapiens	30	14	1	1	1	1	21.	10	0.2
53	sp|P61158|AR	Actin-related protein 3 OS=Homo sapiens OX=9606 GN=ACTR3	30	47	1	1	1	1	3.3	5.	0.0
54	sp|Q00688|FK	Peptidyl-prolyl cis-trans isomerase FKBP3 OS=Homo sapiens	30	25	1	1	1	1	4.9	9.	0.1
55	sp|P60468|SC6	Protein transport protein Sec61 subunit beta OS=Homo sapiens	30	10	1	1	1	1	10.	11	0.3
56	sp|Q07666|KH	KH domain-containing, RNA-binding, signal transduction-	30	48	2	2	2	2	4.5	8.	0.1
57	sp|Q8IYT8|UL	Serine/threonine-protein kinase ULK2 OS=Homo sapiens	30	11	1	1	1	1	0.8	8.	0.0

58	sp|Q6UN15|FI	Pre-mRNA 3~-end-processing factor FIP1 OS=Homo sapiens	30	66	1	1	1	1	2.4	5.	0.0
59	sp|Q75NE6|MI	Putative microRNA 17 host gene protein OS=Homo sapiens	30	83	2	2	1	1	17.	9.	0.4
60	sp|Q9NQ39|R	Putative 40S ribosomal protein S10-like OS=Homo sapiens	30	20	1	1	1	1	3.4	10	0.1
61	sp|A0AV96|R	RNA-binding protein 47 OS=Homo sapiens OX=9606	30	64	1	1	1	1	3.4	7.	0.0
62	sp|Q8N684|CP	Cleavage and polyadenylation specificity factor subunit 7	29	52	1	1	1	1	2.8	7.	0.0
63	sp|Q9Y6Z5|AF	Putative uncharacterized protein AFDN-DT OS=Homo sapiens	28	26	1	1	1	1	3.1	11	0.1
64	sp|Q99567|NU	Nuclear pore complex protein Nup88 OS=Homo sapiens	28	84	1	1	1	1	1.8	5.	0.0
65	sp|Q9H2H9|S3	Sodium-coupled neutral amino acid transporter 1 OS=Homo	28	54	1	1	1	1	2.3	6.	0.0
66	sp|Q9C0B5|Z	Palmitoyltransferase ZDHHC5 OS=Homo sapiens OX=9606	28	78	1	1	1	1	2.2	9.	0.0
67	sp|Q15427|SF3	Splicing factor 3B subunit 4 OS=Homo sapiens OX=9606	27	44	1	1	1	1	3.3	8.	0.0
68	sp|O60256|KP	Phosphoribosyl pyrophosphate synthase-associated protein 2	27	41	1	1	1	1	2.4	7.	0.0
69	sp|P05023|AT1	Sodium/potassium-transporting ATPase subunit alpha-1	27	11	1	1	1	1	1.5	5.	0.0
70	sp|Q86V85|GP	Integral membrane protein GPR180 OS=Homo sapiens OX=9606	27	50	1	1	1	1	3.4	7.	0.0
71	sp|Q9H4K7|M	Mitochondrial ribosome-associated GTPase 2 OS=Homo sapiens	26	44	1	1	1	1	3.4	9.	0.0
72	sp|O75396|SC	Vesicle-trafficking protein SEC22b OS=Homo sapiens OX=9606	26	24	1	1	1	1	4.7	6.	0.1
73	sp|Q9NUQ6|S	SPATS2-like protein OS=Homo sapiens OX=9606 GN=SPATS2L	26	62	1	1	1	1	1.8	9.	0.0
74	sp|Q96PE1|A	Adhesion G protein-coupled receptor A2 OS=Homo sapiens	26	14	1	1	1	1	0.9	8.	0.0
75	sp|Q13573|SN	SNW domain-containing protein 1 OS=Homo sapiens OX=9606	26	61	1	1	1	1	2.8	9.	0.0
76	sp|P49023|PA	Paxillin OS=Homo sapiens OX=9606 GN=PXN PE=1 SV=3	26	65	1	1	1	1	2.9	5.	0.0
77	sp|P46779|RL2	60S ribosomal protein L28 OS=Homo sapiens OX=9606	26	15	1	1	1	1	5.8	12	0.2
78	sp|Q9UKA9|P	Polypyrimidine tract-binding protein 2 OS=Homo sapiens	25	57	1	1	1	1	1.1	8.	0.0
79	sp|P61962|DC	DDB1- and CUL4-associated factor 7 OS=Homo sapiens	25	39	1	1	1	1	3.2	5.	0.0
80	sp|Q9BZE1|R	39S ribosomal protein L37, mitochondrial OS=Homo sapiens	24	48	1	1	1	1	3.8	8.	0.0
81	sp|O96000|ND	NADH dehydrogenase [ubiquinone] 1 beta subcomplex subunit	24	21	1	1	1	1	6.4	8.	0.1
82	sp|Q01844|EW	RNA-binding protein EWS OS=Homo sapiens OX=9606	24	68	1	1	1	1	2.1	9.	0.0
83	sp|Q6ZN19|Z	Zinc finger protein 841 OS=Homo sapiens OX=9606	24	95	1	1	1	1	1	9.	0.0
84	sp|P42694|HE	Probable helicase with zinc finger domain OS=Homo sapiens	24	22	1	1	1	1	0.3	7.	0.0
85	sp|P40222|TX	Alpha-taxilin OS=Homo sapiens OX=9606 GN=TXLNA PE=1	24	62	1	1	1	1	5.1	6.	0.0
86	sp|O94776|MT	Metastasis-associated protein MTA2 OS=Homo sapiens OX=9606	24	75	1	1	1	1	2.4	9.	0.0
87	sp|Q6PJG6|B	BRCA1-associated ATM activator 1 OS=Homo sapiens OX=9606	24	89	1	1	1	1	1.3	5.	0.0
88	sp|Q4G0X9|C	Coiled-coil domain-containing protein 40 OS=Homo sapiens	24	13	1	1	1	1	1	5.	0.0
89	sp|O60318|GA	Germinal-center associated nuclear protein OS=Homo sapiens	24	22	1	1	1	1	1.1	5.	0.0
90	sp|P04275|VW	von Willebrand factor OS=Homo sapiens OX=9606 GN=VWF	23	32	1	1	1	1	0.3	5.	0.0
91	sp|Q96JQ0|PC	Protocadherin-16 OS=Homo sapiens OX=9606 GN=DCHS1	23	34	1	1	1	1	0.3	4.	0.0
92	sp|Q96PB1|CA	N-acetylneuraminate 9-O-acetyltransferase OS=Homo sapiens	23	92	1	1	1	1	1.8	9.	0.0
93	sp|O15243|OB	Leptin receptor gene-related protein OS=Homo sapiens OX=9606	22	14	1	1	1	1	9.9	6.	0.2
94	sp|Q14192|FH	Four and a half LIM domains protein 2 OS=Homo sapiens	20	34	1	1	1	1	4.3	7.	0.1
95	sp|O60884|DN	DnaJ homolog subfamily A member 2 OS=Homo sapiens	20	46	1	1	1	1	2.4	6.	0.0
96	sp|Q86YR7|M	Probable guanine nucleotide exchange factor MCF2L2	19	12	1	1	1	1	1.3	6	0.0
97	sp|Q9C0H6|K	Kelch-like protein 4 OS=Homo sapiens OX=9606 GN=KLHL4	19	81	1	1	1	1	2.5	6.	0.0
98	sp|Q6ZRU5|Y	Putative uncharacterized protein FLJ46089 OS=Homo sapiens	19	17	1	1	1	1	10.	9.	0.1
99	sp|Q8N9W4|G	Golgin subfamily A member 6-like protein 2 OS=Homo sapiens	19	10	1	1	1	1	1	4.	0.0
100	sp|Q86X53|ER	Glutamate-rich protein 1 OS=Homo sapiens OX=9606	19	49	1	1	1	1	3.2	4.	0.0
101	sp|Q9HC52|C	Chromobox protein homolog 8 OS=Homo sapiens OX=9606	17	43	1	1	1	1	1.3	9.	0.0
102	sp|P16070|CD	CD44 antigen OS=Homo sapiens OX=9606 GN=CD44 PE=1	17	82	1	1	1	1	2.2	5.	0.0
103	sp|Q96PM5|Z	RING finger and CHY zinc finger domain-containing protein 1	17	31	1	1	1	1	4.6	6.	0.1
104	sp|Q8NGJ1|O	Olfactory receptor 4D6 OS=Homo sapiens OX=9606 GN=OR4D6	14	36	1	1	1	1	2.2	8.	0.0

Supplementary Table S3. Clinical characteristics of NPC patients according to high and low circWDR37 expression

**Characteristics**	**No. of patients**	**Expression of circWDR37**		***P* value**
		**Low, n (%)**	**High, n (%)**	
**Age**				
< 45	81	58 (57.4)	23 (60.0)	0.868
≥ 45	59	43(42.6)	16 (41.0)	
**Sex**				
Male	24	20 (19.8)	4 (10.3)	0.179
Female	116	81 (80.2)	35(89.7)	
**pre-EBV**				
< 2 (×1000)	59	44 (43.6)	15 (38.5)	0.584
≥ 2 (×1000)	81	57 (56.4)	24 (61.5)	
**T Stage**				
T1-T2	18	11 (10.9)	7 (17.9)	0.263
T3-T4	122	90 (89.1)	32 (82.1)	
**N Stage**				
N0-N1	71	56 (55.4)	15 (38.5)	0.072
N2-N3	69	45 (44.6)	24 (61.5)	
**Death**				
No	115	87 (86.1)	28 (71.8)	**0.047**
Yes	25	14(13.9)	11 (28.2)	
**Distant metastasis (DM)**				
No	114	87(86.1)	27 (69.2)	**0.021**
Yes	26	14(13.9)	12 (30.8)	

Abbreviations: pre-EBV, pretreatment plasma Epstein-Barr virus DNA; OS: overall survival; DFS, disease-free survival; *P* value was determined by χ^2 or Fisher's exact tests.

Supplementary Table S4. Univariate and multivariable Cox regression analysis of circWDR37 expression level and survival in NPC patients

Variable	Univariate analysis			Multivariate analysis		
	**HR**	**95% *CI***	***P* value**	**HR**	**95% *CI***	***P* value**
**Overall survival**						
CircWDR37 expression (high vs. low)	2.357	1.068-5.203	**0.034**	2.531	1.132-5.659	**0.024**
T stage (T3-T4 vs. T1-T2)	1.773	0.418-7.519	0.437			
N stage (N2-N3 vs. N0-N1)	3.651	1.513-8.807	**0.004**	1.943	1.243-3.039	**0.004**
Age (≥ 45 vs. < 45 years)	2.791	1.232-6.320	**0.014**	3.000	1.317-6.834	**0.009**
Gender (Female vs. male)	1.193	0.409-3.479	0.746			
pre-EBV (≥2000 vs. <2000)	9.376	2.210-39.786	**0.002**	6.576	1.524-28.373	**0.012**
**Disease-free survival**						
CircWDR37 expression (high vs. low)	3.018	1.582-5.760	**0.001**	3.324	1.723-6.413	**<0.001**
T stage (T3-T4 vs. T1-T2)	1.286	0.455-3.630	0.635			
N stage (N2-N3 vs. N0-N1)	3.763	1.813-7.811	**< 0.001**	3.827	1.784-8.208	**0.001**
Age (≥ 45 vs. < 45 years)	1.785	0.935-3.408	0.079			
Gender (Female vs. male)	1.449	0.564-3.719	0.441			
pre-EBV (≥2000 vs. <2000)	5.506	2.144-14.137	**< 0.001**	4.208	1.620-10.928	**0.003**
**Distant metastasis-free survival**						
CircWDR37 expression (high vs. low)	2.615	1.208-5.659	**0.015**	2.305	1.062-4.999	**0.035**
T stage (T3-T4 vs. T1-T2)	0.814	0.281-2.364	0.706			
N stage (N2-N3 vs. N0-N1)	4.653	1.853-11.684	**0.001**	3.520	1.388-8.926	**0.008**
Age (≥ 45 vs. < 45 years)	1.741	0.804-3.767	0.159			
Gender (Female vs. male)	1.787	0.536-5.955	0.344			
pre-EBV (≥2000 vs. <2000)	4.489	1.546-13.034	**0.006**	3.457	1.177-10.150	**0.024**

Abbreviations: pre-EBV, pre-treatment plasma Epstein-Barr virus DNA; HR, hazard ratio; NS, not significant. Bold values indicate *P*< 0.05, *P* value was determined by Cox regression analysis.

Supplementary Table S5. Primers and RNA sequences used in this study

**List of oligonucleotide sequences**	**5'→ 3'** (F, forward; R, reverse.)
**Primers for RT-qPCR**	
CircWDR37-F/Divergent	GGCTCTCACTGCTTCTGGAG
CircWDR37-R/Divergent	GCTCTCGATGTCGTGGTCTT
Convergent-circWDR37-F	ACCACGACATCGAGAGCTG
Convergent-circWDR37-R	TCTTGGCCACGCTGACATC
WDR37-F	CCCACAGAAAGCGCAAGTTG
WDR37-R	AACCGAGGAAGGCAGTTTAGA
GAPDH-F/Convergent	AACGGATTTGGTCGTATTGG
GAPDH-R/Convergent	TTGATTTTGGAGGGATCTCG
Divergent-GAPDH-F	CCAATACGACCAAATCCGTT
Divergent-GAPDH-R	CGAGATCCCTCCAAAATCAA
GAPDH-F	GTCTCCTCTGACTTCAACAGCG
GAPDH-R	ACCACCCTGTTGCTGTAGCCAA
β-actin-F	CACCATTGGCAATGAGCGGTTC
β-actin-R	AGGTCTTTGCGGATGTCCACGT
PKR-F	GAAGTGGACCTCTACGCTTTGG
PKR-R	TGATGCCATCCCGTAGGTCTGT
U6-F	CTCGCTTCGGCAGCACAT
U6-R	TTTGCGTGTGTCATCCTTGCG
**shRNAs**	
sh-circWDR37-F	CTAGTATTGTCTCCAGCTT
sh-circWDR37-R	AAGCTGGAGACAATACTAG
**siRNAs**	
si-circWDR37-1	GTTGCTGACACTAGTATTG
si-circWDR37-3	CTAGTATTGTCTCCAGCTT
si-PKR-564	GGUGAUCUUUCAGCAGGUUTT
si-PKR-2058	GGCAUCAUCUCAGAUAUAUTT

* F, forward; R, reverse.

Supplementary Table S6. Antibodies used in this study

Antibodies	Source	Identifier
Anti-IKKβ	Cell Signaling Technology	2678T
Anti-p-IKKα/β	Cell Signaling Technology	2697T
Anti-p65	Proteintech	10745-1-AP
Anti-p-p65	Cell Signaling Technology	3033T
Anti-IκBα	Cell Signaling Technology	4814T
Anti-p-IκBα	Cell Signaling Technology	2859T
Anti-PKR	Proteintech	18244-1-AP
Anti-p-PKR-T446	Abcam	ab32036
Anti-p-PKR-T451	Abcam	ab81303
Anti-GAPDH	Abcam	ab8245
Anti-α-tubulin	Proteintech	66031-1-Ig
Anti-eIF2α	Beyotime	AF6771
Anti-p-eIF2α	Beyotime	AF1237
Anti-Flag	Sigma-Aldrich	F1804
Anti-HA	Proteintech	51064-2-AP
Anti-myc	Proteintech	16286-1-AP
Anti-$p16^{INK4A}$	Proteintech	10883-1-AP
Anti-Cyclin D1	Proteintech	60186-1-Ig
